# Anticancer Activity and Cisplatin Binding Ability of *Bis*-Quinoline and *Bis*-Isoquinoline Derived [Pd_2_L_4_]^4+^ Metallosupramolecular Cages

**DOI:** 10.3389/fchem.2018.00563

**Published:** 2018-11-22

**Authors:** Roan A. S. Vasdev, Lachlan F. Gaudin, Dan Preston, Jackmil P. Jogy, Gregory I. Giles, James D. Crowley

**Affiliations:** ^1^Department of Chemistry, University of Otago, Dunedin, New Zealand; ^2^MacDiarmid Institute for Advanced Materials and Nanotechnology, Wellington, New Zealand; ^3^Department of Pharmacology and Toxicology, University of Otago, Dunedin, New Zealand

**Keywords:** palladium(II), anticancer, self-assembly, metallosupramolecular, quinoline

## Abstract

New *bis-*quinoline (**L**_q_) and *bis-*isoquinoline-based (**L**_iq_) ligands have been synthesized, along with their respective homoleptic [Pd_2_(**L**_q_ or **L**_iq_)_4_]^4+^ cages (**C**_q_ and **C**_iq_). The ligands and cages were characterized by ^1^H, ^13^C and diffusion ordered (DOSY) NMR spectroscopies, high resolution electrospray ionization mass spectrometry (HR-ESIMS) and in the case of the *bis*-quinoline cage, X-ray crystallography. The crystal structure of the **C**_q_ architecture showed that the [Pd_2_(**L**_q_)_4_]^4+^ cage formed a twisted *meso* isomer where the [Pd(**quinoline**)_4_]^2+^ units at either end of the cage architecture adopt the opposite twists (left and right handed). Conversely, Density Functional Theory (DFT) calculations on the **C**_iq_ cage architecture indicated that a lantern shaped conformation, similar to what has been observed before for related [Pd_2_(**L**_tripy_)_4_]^4+^ systems (where **L**_tripy_ = 2,6-*bis*(pyridin-3-ylethynyl)pyridine), was generated. The different cage conformations manifest different properties for the isomeric cages. The **C**_iq_ cage is able to bind, weakly in acetonitrile, the anticancer drug cisplatin whereas the **C**_q_ architecture shows no interaction with the guest under the same conditions. The kinetic robustness of the two cages in the presence of Cl^−^ nucleophiles was also different. The **C**_iq_ cage was completely decomposed into free **L**_iq_ and [Pd(Cl)_4_]^2−^ within 1 h. However, the **C**_q_ cage was more long lived and was only fully decomposed after 7 h. The new ligands (**L**_iq_ and **L**_q_) and the Pd(II) cage architectures (**C**_iq_ and **C**_q_) were assessed for their cytotoxic properties against two cancerous cell lines (A549 lung cancer and MDA-MB-231 breast cancer) and one non-cancerous cell line (HDFa skin cells). It was found that **L**_q_ and **C**_q_ were both reasonably cytotoxic (IC_50S_ ≈ 0.5 μM) against A549, while **C**_iq_ was slightly less active (IC_50_ = 7.4 μM). **L**_iq_ was not soluble enough to allow the IC_50_ to be determined against either of the two cancerous cell lines. However, none of the molecules showed any selectivity for the cancer cells, as they were all found to have similar cytotoxicities against HDFa skin cells (IC_50_ values ranged from 2.6 to 3.0 μM).

## Introduction

Metallosupramolecular architectures (MSAs) (Cook and Stang, 2015) have been attracting increasing attention over the past two decades due to their many potential applications including catalysis (Yoshizawa et al., 2009; Yoshizawa and Fujita, 2010; Martí-Centelles et al., 2018), storage (Mal et al., 2009), and sensing (Wang et al., 2011). Inspired by the success of cisplatin and other metallodrugs (Mjos and Orvig, 2014) there is emerging interest in exploiting MSAs for biomedical purposes (Cook et al., 2013; Therrien, 2015; Casini et al., 2017; Zhou et al., 2017). Several groups have examined MSAs as drug delivery vectors (Therrien et al., 2008; Schmitt et al., 2012; Yi et al., 2012; Zheng et al., 2015; Samanta et al., 2016, 2017; Bhat et al., 2017; Xu et al., 2017; Wang J.-F. et al., 2018; Yue et al., 2018). Additionally, MSAs have been shown to bind DNA (Oleksy et al., 2006; Garci et al., 2017; Zhao et al., 2017) and RNA (Phongtongpasuk et al., 2013; Malina et al., 2015), interact with proteins (Li et al., 2014; Mitchell et al., 2017) and have anticancer (Hotze et al., 2008; Faulkner et al., 2014; Grishagin et al., 2014; Dubey et al., 2015; Zheng et al., 2016; Allison et al., 2018) and antibacterial (Richards et al., 2009; Howson et al., 2012; Wang H. et al., 2018) properties.

Since the pioneering work of McMorran and Steel (McMorran and Steel, 1998) interest in [M_2_(**L**)_4_]^n+^ cage-type structures has burgeoned (Schmidt et al., 2014). Some time ago now we reported that a [Pd_2_(**L**_tripy_)_4_]^4+^ cage (where **L**_tripy_ = 2,6-*bis*(pyridin-3-ylethynyl)pyridine) could host two molecules of the anticancer drug cisplatin within the cavity of the cage (Lewis et al., 2012), and thus had potential as a drug delivery vector. Disappointingly, the binding event, which was governed mainly by hydrogen bonding interactions, was weak (Preston et al., 2015). The host-guest complex formed in acetonitrile (CH_3_CN) and dimethylformamide (DMF) but unfortunately, in more hydrogen bond competitive solvents such as water and dimethyl sulfoxide (DMSO) no host-guest interaction was observed. Additionally, the parent Pd(II) based cage decomposed rapidly in the presence of nucleophiles (Lewis et al., 2012; McNeill et al., 2015). Thus, in order to exploit these [Pd_2_(**L**)_4_]^4+^ cages as cisplatin delivery vehicles these issues need to be addressed. We and others have examined a range of modifications to the parent [Pd_2_(**L**)_4_]^4+^ cage system in order to improve the solubility (Lewis and Crowley, 2014; Preston et al., 2015; Han et al., 2017) and other properties (Lewis et al., 2013, 2014; Kaiser et al., 2016; Schmidt et al., 2016a) of the cage. Efforts have also been made to enhance the strength of the host-guest interaction (Kim et al., 2015) and the stability of the cages in the presence of biologically revelant nucleophiles (Preston et al., 2016). However, while some improvements have been made these [Pd_2_(**L**)_4_]^4+^ cages still require further modifcations in order to be useful drug delivery vectors.

The [Pd_2_(**L**)_4_]^4+^ cages have also been examined for their cytotoxic properties. We showed that the parent [Pd_2_(**L**_tripy_)_4_]^4+^ was modestly cytotoxic (IC_50_ values range from 40 to 70 μM) against a range of cancer cell lines but was less active than related bis-1,2,3-triazole [Pd_2_(**L**)_4_]^4+^ helicates (McNeill et al., 2015). We also examined the cytotoxicity of related amino substituted [Pd_2_(**L**)_4_]^4+^ cages against the same panel of cancer cells and found that they exhibited similar cytotoxic properties as the parent systems (Preston et al., 2016). Casini, Kühn and co-workers (Kaiser et al., 2016; Schmidt et al., 2016b) have measured the cytotoxicity of a series of related [Pd_2_(**L**)_4_]^4+^ cages and observed similar IC_50_ values (10–70 μM). Additionally, they have measured the cytotoxicity of mixtures of the cages and cisplatin and unsurprisingly have found that those mixtures are more cytotoxic than cage alone (IC_50_ = 2–13 μM). Yoshizawa, Ahmedova and co-workers have also found that [M_2_(**L**)_4_]^4+^ (M = Pd^2+^ or Pt^2+^) cages with similar, but more hydrophobic, dipyridyl anthracenyl ligands (**L**_anthracene_) display high anticancer activity (IC_50_ values range from 0.9 to 37.4 μM against HL-60, HL-60/Dox, HT-29, T-24, SKW-3 cancer cell lines) (Ahmedova et al., 2016; Anife et al., 2016).

The majority of the [Pd_2_(**L**)_4_]^4+^ cages examined to date feature pyridyl donors, as part of our efforts to improve the biological properties of these systems herein we describe the use of isoquinoline and quinoline-derived ligands for the assembly of two new [Pd_2_(**L**)_4_]^4+^ cages. While is well-known that isoquinoline and quinoline ligands can bind with palladium(II) and platinum(II) (Bondy et al., 2004) their use as donor systems in ligands for the generation MSAs has not been extensively explored (Bloch et al., 2016, 2017). These quinoline derived systems feature different electronic and steric properties compared to the parent pyridyl systems thus we also examine the effect these changes have on the host-guest chemistry with cisplatin, the stability of the cages in the presence of nucleophiles and the antiproliferative properties of the cages.

## Results and discussion

**Figure 1:**
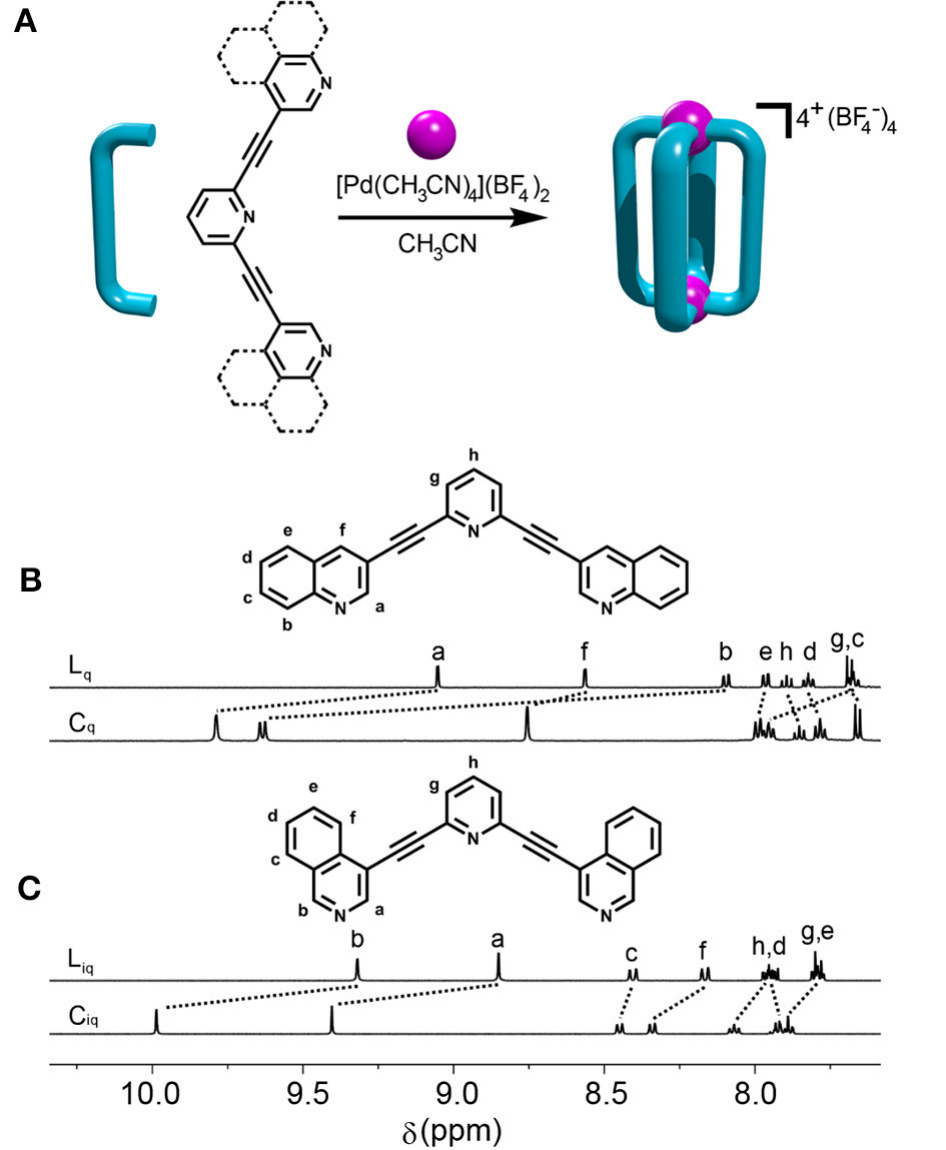
**(A)** General scheme for formation of [Pd_2_(**L**)_4_]^4+^ cages (**C**_**q**_ and **C**_**iq**_) and partial ^1^H NMR spectra (400 MHz, CD_3_CN, 298 K) of **(B) L**_**q**_ and **C**_**q**_, and **(C) L**_**iq**_ and **C**_**iq**_.

The synthesis of the new quinoline (**L**_q_) and isoquinoline (**L**_iq_) based ligands was facile (Supplementary Material). Using sequential Sonogashira carbon-carbon cross coupling reactions from commercially available building blocks the ligands were generated in good yields (**L**_q_ = 86% and **L**_iq_ = 78%). ^1^H and ^13^C NMR spectroscopic data were consistent with the formation of the ligands which was supported by high-resolution electrospray mass spectrometry (HR-ESIMS) (Figure 1 and Supplementary Material). Peaks corresponding to protonated and sodiated ligand were observed at *m/z* = 382.1320 and 404.1132, respectively, for **L**_iq_ and similar peaks were observed for **L**_q_ (Supplementary Material).

With the ligands in hand, the complexation with [Pd(CH_3_CN)_4_](BF_4_)_2_ in acetonitrile was examined (Figure 1). The cage formation was monitored using ^1^H NMR spectroscopy (CD_3_CN, 298 K) and showed that mixing [Pd(CH_3_CN)_4_](BF_4_)_2_ and the **L**_iq_ ligand at room temperature (RT) in a 1:2 ratio led to the rapid (< 2 min) and quantitative formation of the expected **C**_iq_ cage (Figure 1), similar to what was observed with the parent **L**_tripy_ system (Lewis et al., 2012). Interestingly, the behavior of **L**_q_with [Pd(CH_3_CN)_4_](BF_4_)_2_ at room temperature was very different. After 5 min at RT the reaction mixture displayed multiple proton resonances, none of which were due to free ligand, consistent with the formation of a mixture of different cage isomers. The reaction was monitored using ^1^H NMR spectroscopy for 24 h at RT however little to no changes were observed after the first hour and the spectrum still displayed multiple proton resonances. A ^1^H DOSY experiment (CD_3_CN, 298 K) on the mixture showed that all the different proton resonances had the same diffusion co-efficient consistent with the postulate that the reaction mixture contains a series of cage isomers (Supplementary Material).

**Figure 2:**
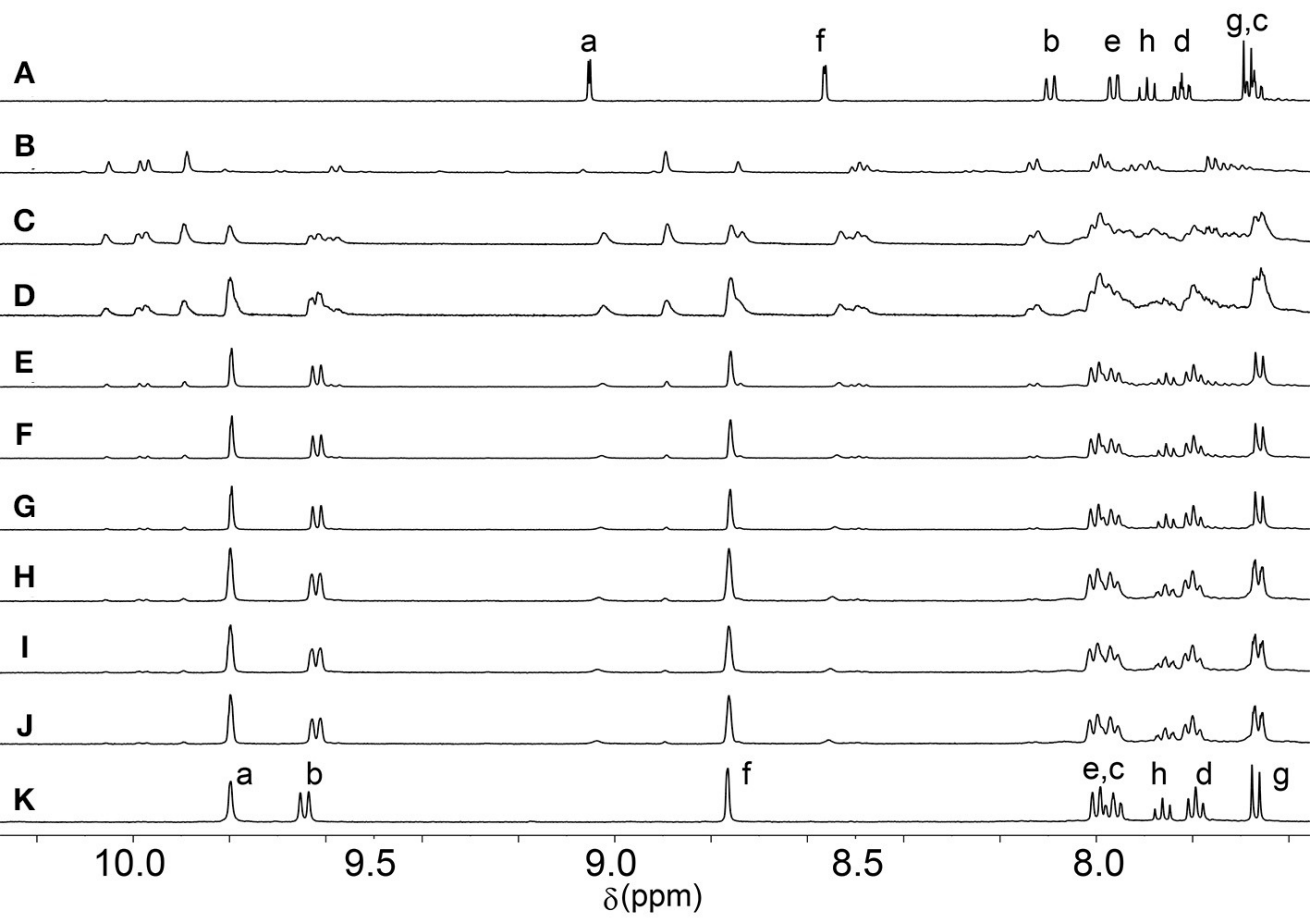
Partial ^1^H NMR (500 MHz, CD_3_CN, 338 K) spectra showing the formation of **C**_**q**_ over time at 65°C. **(A) L**_**q**_, **(B)** initial complexation (*t* = 0), **(C)** *t* = 30 min, **(D)** *t* = 1 h, **(E)** *t* = 2 h, **(F)** *t* = 3 h, **(G)** *t* = 4 h, **(H)** *t* = 5 h, **(I)** *t* = 6 h, **(J)** *t* = 7 h, **(K)** isolated **C**_**q**_.

The assembly reaction between **L**_q_with [Pd(CH_3_CN)_4_](BF_4_)_2_ was then carried out at 65°C, in CD_3_CN and again monitored using ^1^H NMR spectroscopy (Figure 2). After 5 min the same complicated series of proton resonance were observed. However, with continued heating this slowly resolved into a single series of resonances (after 7 h), consistent with the formation of a single cage isomer (Figure 2). Pleasingly, both cages (**C**_iq_ and **C**_q_) could be isolated by adding diethyl ether into the acetonitrile reaction mixtures providing the cages as colorless/tan precipitates in 88% (**C**_iq_) or 92% (**C**_q_) yield, respectively. ^1^H NMR spectroscopy (CD_3_CN, 298 K) exhibited the expected downfield shifts of the signals pertaining to protons H_a_, H_b_ and H_f_ as well as the anticipated downfield shifts of the rest of the isoquinoline and quinoline protons resonances (Figure 1). HR-ESIMS data also supported the formation of the cages, showing ions corresponding to the loss of 2, 3 and 4 tetrafluoroborate (${\text{BF}}_{4}^{-}$) counterions (*m/z* = 956.1610 (2^+^), 608.4424 (3^+^), and 434.5832 (4^+^), Supplementary Material). ^1^H DOSY experiments (CD_3_CN, 298 K) on the ligands (Diffusion coefficients (D) = 13.1 (**L**_q_) and 15.0 (**L**_iq_) x 10^−10^ m^2^ s^−1^) and cages (D = 4.1 (**C**_q_) and 4.3 (**C**_iq_) × 10^−10^ m^2^ s^−1^ were also consistent with the formation of the larger [Pd_2_(**L**)_4_]^4+^ cages (Supplementary Material).

**Figure 3:**
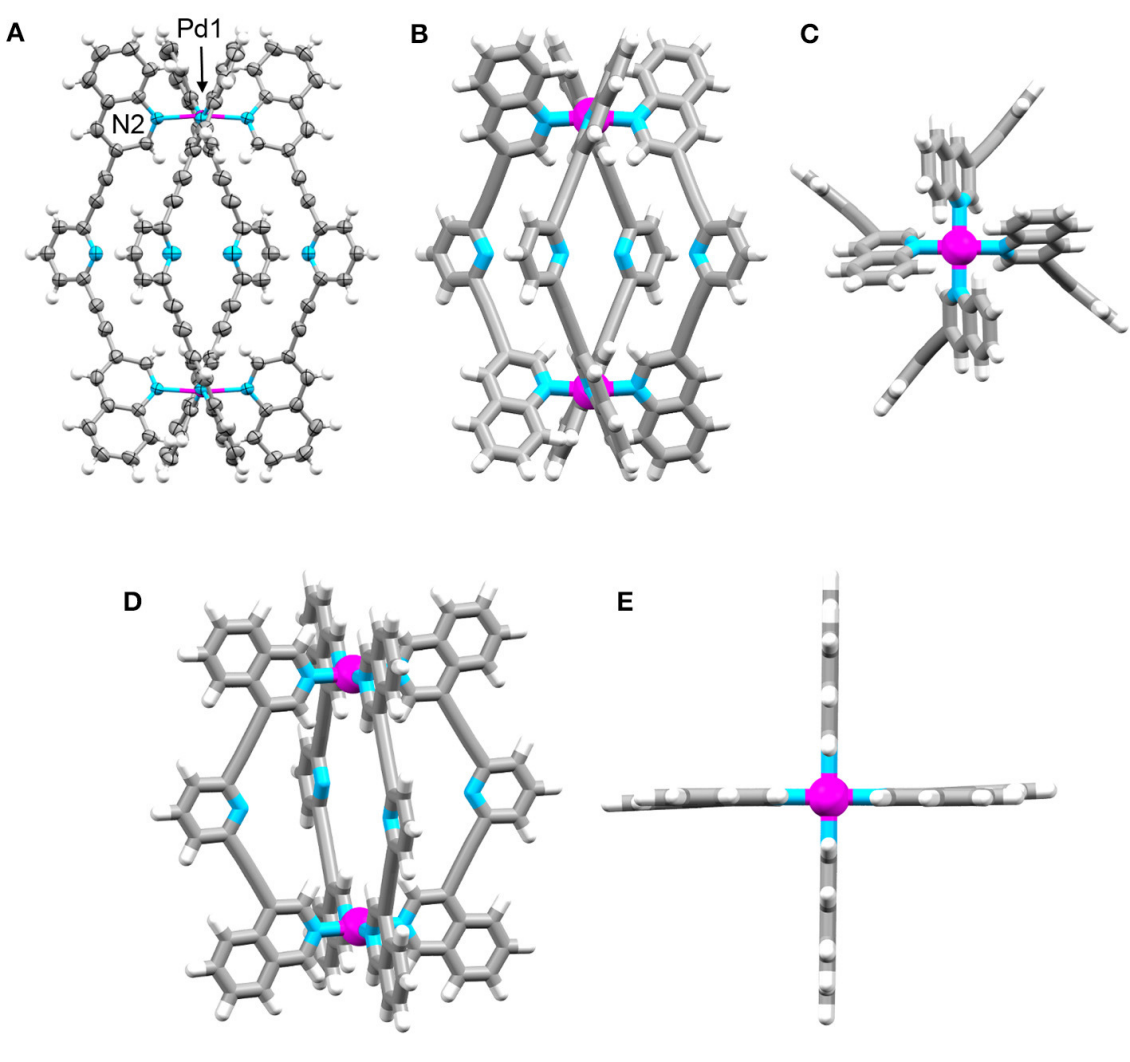
Molecular structures of **C**_**q**_ and **C**_**iq**_. X-ray structure of **C**_**q**_: **(A)** ellipsoid side view, **(B)** tube side view, and **(C)** tube top view showing paddle-like array of quinoline panels over palladium(II) center. Solvent molecules and counterions have been omitted for clarity. Ellipsoids are shown at 50% probability. DFT optimized (BP86 def2-SVP) model of **C**_**iq**_; **(D)** side view and **(E)** top view showing lantern-shaped structure. Colors: carbon gray, nitrogen blue, palladium magenta, hydrogen white.

Crystals of **C**_q_ suitable for X-ray diffraction formed during the cooling of an acetonitrile solution of the cage from 65°C to room temperature. The structure was solved in the tetragonal space group P4/*mnc* with the asymmetric unit containing one eighth of the cage and one quarter of a ${\text{BF}}_{4}^{-}$ counterion (Figure 3 and Supplementary Material). The other ${\text{BF}}_{4}^{-}$ anions and some acetonitrile molecules could not be modeled sensibly thus the SQUEEZE routine was employed to account for the diffuse electron density (Supplementary Material). The data revealed the expected [Pd_2_(**L**_q_)_4_]^4+^ cage structure. The Pd-N bond lengths (Pd1-N2 2.045 Å) were similar to what have been previously observed for the related [Pd_2_(**L**_tripy_)_4_]^4+^ cages where the Pd-N bond lengths range from 2.016 to 2.027 Å (Lewis et al., 2012; Lewis and Crowley, 2014). The **L**_q_ ligands of the cage are twisted giving a V-shaped conformation where the terminal quinoline and central pyridyl heterocyclic units are not co-planar which is quite different to what was observed with the [Pd_2_(**L**_tripy_)_4_]^4+^ cages. In X-ray structures of the parent [Pd_2_(**L**_tripy_)_4_]^4+^ cages the **L**_tripy_ ligands were found in a linear conformation with the heterocyclic units coplanar. The twisting also alters the Pd1-Pd1′ distance within **C**_q_ related to the [Pd_2_(**L**_tripy_)_4_]^4+^ cages. The Pd1-Pd1′ distances for the parent [Pd_2_(**L**_tripy_)_4_]^4+^ cages range from 11.49 to 12.24 Å, whereas the Pd1-Pd1′ distance was found to be longer (12.506 Å) suggesting that the **C**_q_cage has a larger cavity despite featuring the same 2,6-diethynylpyridine linker units. The [Pd(**quinoline**)_4_]^2+^ units at the top and bottom of **C**_q_ are twisted in opposite directions, the top cationic unit has a right handed twist while the bottom cationic unit has a left handed twist giving an overall *meso* structure (Figures 3B,**C** and Supplementary Material). Despite extensive efforts we were unable to obtain X-ray diffraction quality single crystals of **C**_iq_. Thus, to gain further insight into the structure of **C**_iq_ we modeled the cage using Density Functional Theory (DFT) calculations (Figures 3D,E). Energy minimization of **C**_iq_ (DFT, BP86 def2-SVP, acetonitrile solvation, Supplementary Material) showed that the cage adopted a lantern shape similar to what was previously observed for [Pd_2_(**L**_tripy_)_4_]^4+^ cages (Lewis et al., 2012; Lewis and Crowley, 2014). The calculated Pd – N bond distances (2.049 Å) and the Pd-Pd′ distance (11.758 Å) match well with those observed crystallographically for the related [Pd_2_(**L**_tripy_)_4_]^4+^ cages. The **L**_iq_ ligand adopts a linear conformation with all the heterocyclic units coplanar. The DFT calculations indicated that the **C**_iq_ is structurally very similar to the parent [Pd_2_(**L**_tripy_)_4_]^4+^ cages whereas the **C**_q_ is more twisted and provided a cavity of different size and shape to the parent cages and the **C**_iq_ system.

**Figure 4:**
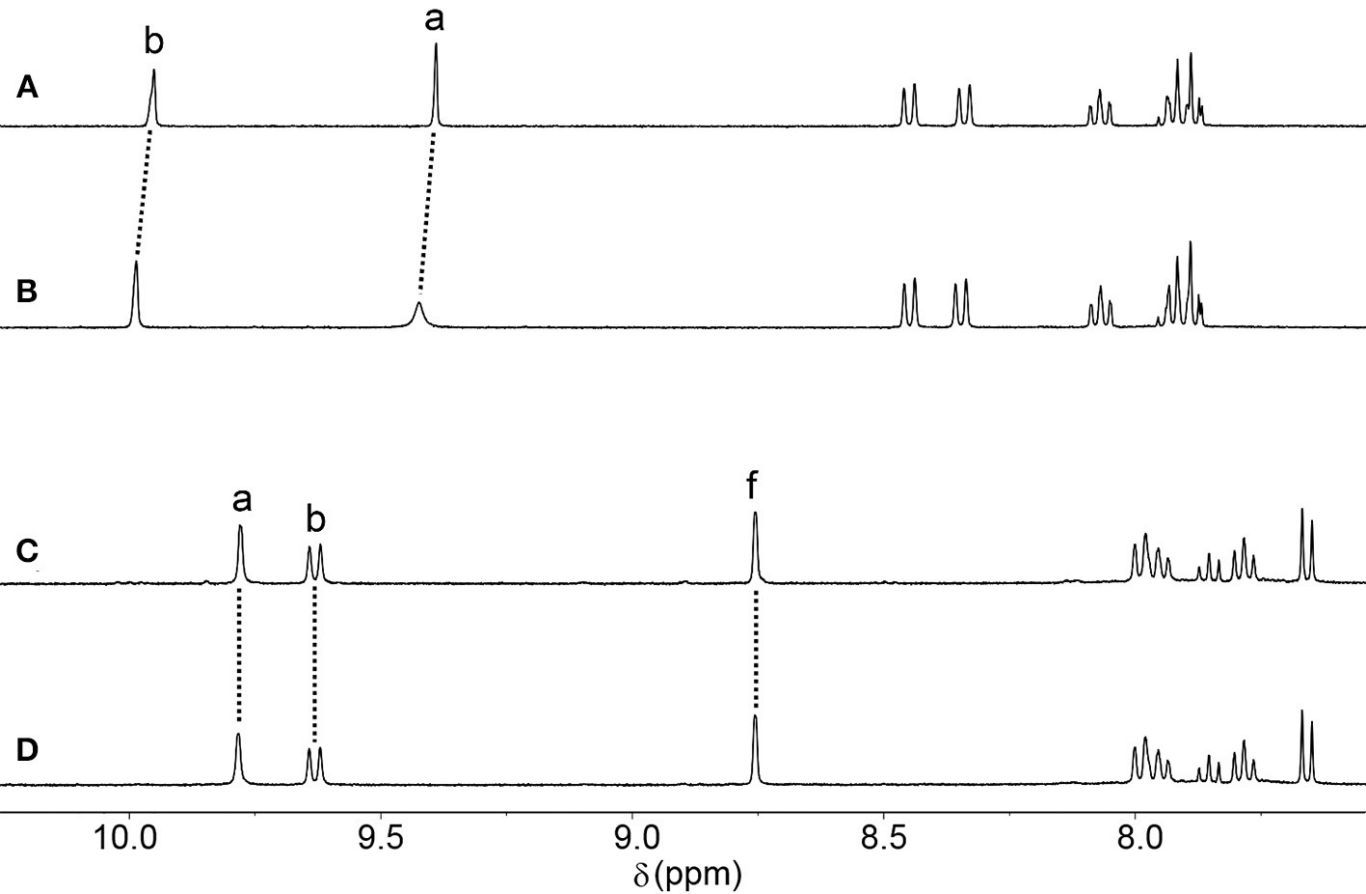
Partial ^1^H NMR (500 MHz, CD_3_CN, 298 K) stacked plot of **(A) C**_**iq**_ **(B) C**_**iq**_ + cisplatin (10 eq.) **(C) C**_**q**_, and **(D) C**_**q**_ + cisplatin (10 eq.).

We and others have previously shown that other similar [Pd_2_(**L**_tripy_)_4_]^4+^ cages can encapsulate cisplatin through hydrogen bonding interactions in CH_3_CN and DMF solvents (Lewis et al., 2012, 2013, 2014; Kaiser et al., 2016; Preston et al., 2016, 2017; Schmidt et al., 2016b). Therefore, we examined the ability of **C**_iq_ and **C**_q_ to interact with cisplatin in CH_3_CN using ^1^H NMR spectroscopy. Addition of an excess of cisplatin to a CD_3_CN solution of the **C**_iq_ cage resulted in a downfield shift and broadening (Δδ = 0.03 ppm) of the internally directed cage proton H_a_ (Figures 4A,B) indicative of cisplatin binding within the cage cavity, albeit weakly. A similar ^1^H NMR experiment was carried out with the **C**_q_ cage (Figures 4C,D). However, with the **C**_q_ cage no shifts were observed for any of the cage proton resonances in the presence of an excess of cisplatin suggesting that the more twisted **C**_q_ cage does not interact with the anticancer agent. The behavior was similar to what has been observed with a related twisted [Pd_2_(**L**_2Atripy_)_4_]^4+^ cage (where **L**_2Atripy_ = 2,6-*bis*[2-(6-amino-3-pyridinyl)ethynyl]-4-pyridinemethanol) (Preston et al., 2016). The [Pd_2_(**L**_2Atripy_)_4_]^4+^ cage did not bind cisplatin in DMF solvent and the lack of binding was ascribed to the twisted cage cavity which was not as preorganised as those of the related lantern shaped [Pd_2_(**L**_tripy_)_4_]^4+^ cages. Presumably the different sized cavity and different spatial arrangement of the hydrogen bond donors and acceptors caused by the twisting observed in the crystal structure of **C**_q_ impedes the cisplatin-**C**_q_ interaction in this case.

**Figure 5:**
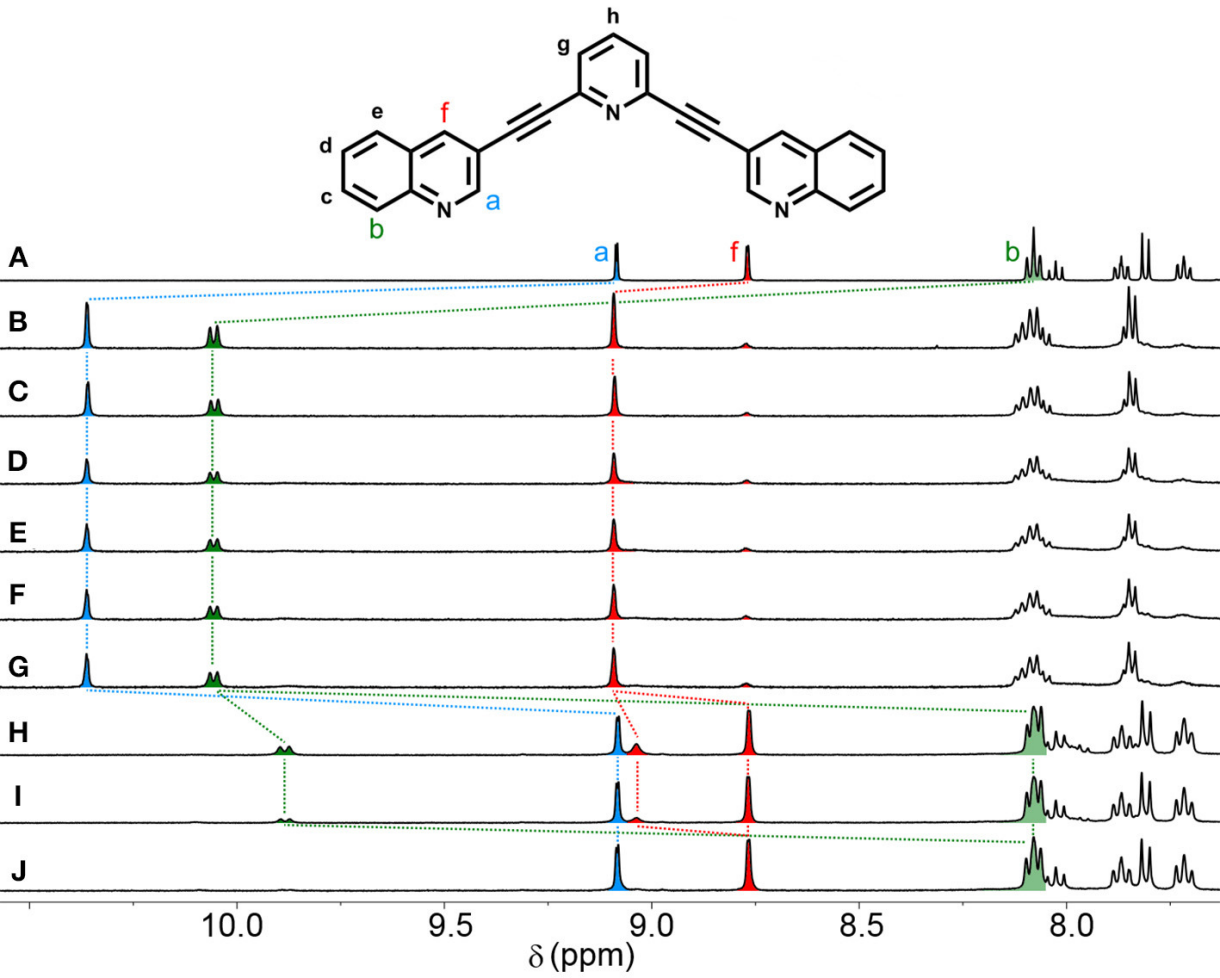
Partial ^1^H NMR (500 MHz, *d*_6_-DMSO, 298 K) spectra showing the stability of **C**_**q**_ in the presence of 8 eq. Cl^−^ anions. **(A) L**_**q**_, **(B) C**_**q**_, **(C) C**_**q**_ with 8 eq. Cl^−^ (*t* = 2 min), **(D)** *t* = 10 min, **(E)** *t* = 20 min, **(F)** *t* = 30 min, **(G)** *t* = 1 h, **(H)** *t* = 3 h, **(I)** *t* = 5 h, **(J)** *t* = 7 h.

The kinetic robustness of the related [Pd_2_(**L**_tripy_)_4_]^4+^ architectures in the presence of common biological nucleophiles (chloride (Cl^−^), histidine and cysteine) has been determined using ^1^H NMR competition experiments. When the parent [Pd_2_(**L**_tripy_)_4_]^4+^ architectures were treated with 8 equivalents of tetrabutylammonium chloride the pyridyl substituted cages were rapidly and quantitatively decomposed (in < 5 min). To examine the effect of substituting the pyridyl donor units for quinoline heterocycles time-course ^1^H NMR competition experiments were carried out in *d*_6_-DMSO where 2 mM solutions of each cage (**C**_q_or **C**_iq_) were treated with 8 equivalents of tetrabutylammonium chloride at 298 K (Figure 5 and Supplementary Material). Within 30 s of adding Cl^−^ to the **C**_iq_ cage, there were multiple species observed in the ^1^H NMR spectrum. These were attributed to the **C**_iq_ cage, [Cl⊂**C**_iq_]^3+^, the [Pd_2_(**L**_iq_)_2_Cl_4_] macrocycle and free ligand based on our own previous results (Preston et al., 2015) and related literature. After 50 min, only uncoordinated ligand was visible in the ^1^H NMR spectrum (Supplementary Material).

Under the same conditions, **C**_q_ was stable for 1 h before showing signs of decomposition (Figure 5). After 3 h, there was no evidence of the **C**_q_ cage, and the ^1^H NMR spectrum displayed peaks corresponding to free ligand and a second metal-containing species, which based on the observed chemical shifts was most likely the neutral [Pd_2_(**L**_q_)_2_Cl_4_] macrocycle (Figure 5H). This degradation behavior has been seen before with the [Pd_2_(**L**_tripy_)_4_]^4+^ system in DMF (Preston et al., 2015). After 7 h, only free ligand could observed in the ^1^H NMR spectrum indicating that all the ligand containing metal complexes had been completely decomposed into [Pd(Cl)_4_]^2−^(Figure 5J).

**Figure 6:**
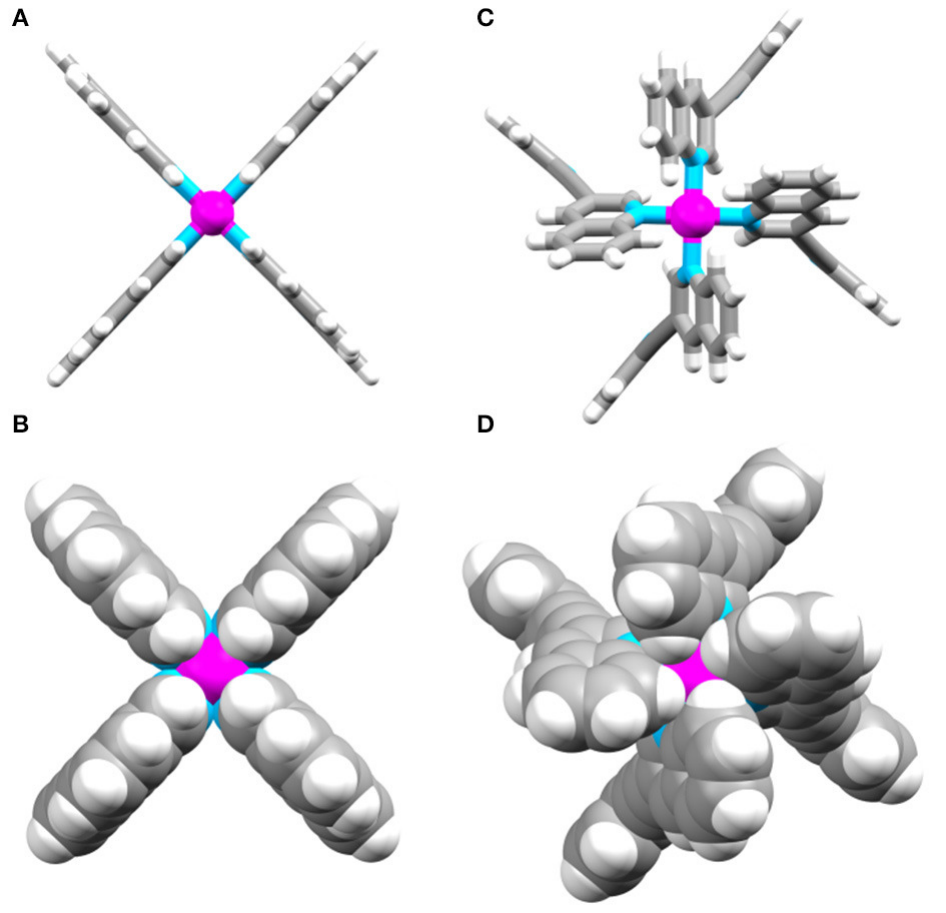
Top down views of **(A)** and **(B)** the DFT optimized model of **C**_**iq**_, and **(C)** and **(D)** the X-ray structure of **C**_**q**_.

In comparison to the previously reported [Pd_2_(**L**_tripy_)_4_]^4+^ cage (τ_1/2_ = 2 min), the isoquinoline cage displayed an identical half-life (τ_1/2_ = 2 min), whereas the quinoline system was considerably more robust (τ_1/2_ = 2 h). Presumably the observed results reflect the different steric profiles of the two quinoline substituted cages (**C**_q_or **C**_iq_). The **C**_q_ cage has the quinoline moieties protecting the external face of the palladium, providing more impediment to nucleophilic attack from that face (Figure 6). The **C**_iq_ does not feature the same steric impediment as the benzene units of the isoquinoline heterocycles do not block the top face of the **C**_iq_ cage as much as they do in the quinoline **C**_q_(Figure 6).

To assess biological activity, the cytotoxic effect (as half-maximal inhibitory concentrations (IC_50_)) of the ligands and cages were determined against three different cell lines: cisplatin resistant MDA-MB-231 (breast cancer) (Lehmann et al., 2011), A549 (lung cancer) and non-cancerous primary cells: adult human dermal fibroblasts (HDFa) (Table 1 and Supplementary Material). The ligands **L**_q_ and **L**_iq_ exhibited limited solubility, and so data above the concentration of 1 μM was unattainable. Below this threshold, **L**_iq_ displayed minimal cytotoxic activity against both cell lines, while **L**_q_ was shown to be cytotoxic against A549 (IC_50_ = 0.5 μM). Both cages were observed to be cytotoxic against the malignant cell lines, with **C**_q_ showing the same level of toxicity as its ligand against lung cancer cells (IC_50_ = 0.5 μM). **C**_q_ was slightly less cytotoxic against MDA-MB-231 (IC_50_ = 1.7 μM), whereas **C**_iq_ was less cytotoxic than the quinoline analog, with the IC_50_ values ranging from 4.0 to 7.4 μM against the cancer cells. Both quinoline cages were found to be considerably more active than the related parent [Pd_2_(**L**_tripy_)_4_]^4+^ cage system (IC_50_ = 41.4 and 56.7 μM against A549 and MDA-MB-231, respectively) (McNeill et al., 2015). The quinoline cages were also more active than cisplatin against the two cancer lines examined (cisplatin IC_50_ values = 41.2 and 9.4 μM, against MDA-MB-231 and A549, respectively) (Lo et al., 2015; McNeill et al., 2015). The quinoline cages **C**_q_ and **C**_iq_ were more cytotoxic than all the [Pd_2_(**L**_tripy_)_4_]^4+^ cage systems reported in the literature (IC_50_ values for the **L**_tripy_ based systems ranged from 10 to 100 μM) (McNeill et al., 2015; Kaiser et al., 2016; Schmidt et al., 2016b). Additionally, **C**_q_ was also more active, albeit against different cancer cell lines (HL-60, HL-60/Dox, HT-29, T-24, SKW-3), than the hydrophobic [Pd_2_(**L**_anthracene_)_4_]^4+^ cages of Yoshizawa and Ahmedova (IC_50_ values ranged from 0.9 to 37.4 μM) (Ahmedova et al., 2016; Anife et al., 2016). **C**_q_ was also more cytotoxic than a hydrophobic *bis*-hexyl-1,2,3-triazole substituted [Pd_2_(**L**_hextrz_)_4_]^4+^ helicate, **C**_hextrz_, we developed previously (IC_50_ values = 6.9 and 6.0 μM against A549 and MDA-MB-231, respectively) (McNeill et al., 2015). We presume that the favorable combination of high hydrophobicity and the kinetic robustness against biological nucleophiles leads to the higher observed activity of **C**_q_ relative to the other [Pd_2_(**L**)_4_]^4+^ architectures. Disappointingly, neither of the cages (**C**_q_ and **C**_iq_) showed any selectivity for the cancer cells, they were all found to have similar cytotoxicity against HDFa skin cells (IC_50_ values ranged from 2.6 to 3.0 μM).

**Table 1:** Half-maximal inhibitory concentrations (IC_50_) of ligands **L**_**q**_ and **L**_**iq**_, and cages **C**_**q**_ and **C**_**iq**_ architectures at 24 h.

**Compound**	**${\text{IC}}_{50}^{\text{a}}$ (μM)**			
	**MDA-MB-231**	**A549**	**HDFa**	**MCF-10A^b^**
**L**_**q**_	>1^c^	0.5 ± 0.1	>1^c^	–
**C**_**q**_	1.7 ± 0.1	0.5 ± 0.1	2.6 ± 0.4	–
**L**_**iq**_	>1^c^	>1^c^	>1^c^	–
**C**_**iq**_	4.0 ± 0.3	7.4 ± 1.0	3.0 ± 0.4	–
**L**_hextrz *(McNeilletal.,2015)*_	89.8 ± 10.7	28.5 ± 2.6	–	18.1 ± 3.1
**C**_hextrz *(McNeilletal.,2015)*_	6.0 ± 0.6	6.9 ± 0.9	–	8.1 ± 1.2
**L**_tripy *(McNeilletal.,2015)*_	>100	95.3 ± 9.7	–	>100
**C**_tripy *(McNeilletal.,2015)*_	56.7 ± 2.2	41.4 ± 3.9	–	71.4 ± 3.9
cisplatin_*(Loetal.,2015; McNeilletal.,2015)*_	41.2 ± 3.9	9.4 ± 0.3	–	–

***L***_**tripy**_, ***C***_**tripy**_, ***L***_**hextrz**_, ***C***_**hextrz**_ and cisplatin (Lo et al., 2015) have been added for comparison (McNeill et al., 2015).

*^a^IC_50_ values are given as mean ± SE*.

b The ***C***_**tripy**_ and ***C***_**hextrz**_ cages were tested against MCF-10A as a non-cancerous control.

c *Solubility limited the range of concentrations to below 1 μM. “ – “ = Not determined*.

## Conclusion

We have herein reported the synthesis, characterization, cisplatin binding, kinetic robustness and cytotoxicity of two new *bis-*isoquinoline and *bis*-quinoline derived [Pd_2_**(L)**_4_]^4+^ cage complexes. The crystal structure of **C**_q_ architecture showed that the [Pd_2_(**L**)_4_]^4+^ cage formed a twisted *meso* isomer where the [Pd(**quinoline**)_4_]^2+^ units at either end of the cage architecture adopt the opposite twists (left and right handed). Conversely, Density Functional Theory (DFT) calculations on the **C**_iq_ cage architecture indicated that a lantern shaped conformation similar to what has been observed before for related [Pd_2_(**L**_tripy_)_4_]^4+^ systems was generated. The different cage conformations resulted in different properties for the isomeric cages. The **C**_iq_ cage is able to bind, weakly in acetonitrile, the anticancer drug cisplatin whereas the **C**_q_ architecture shows no interaction with the guest under the same conditions. The kinetic robustness of the two cages in the presence of Cl^−^ nucleophiles was also different. The **C**_iq_ cage was completely decomposed into free **L**_iq_ and [Pd(Cl)_4_]^2−^within 1 h. However, the **C**_q_ cage was more long lived and was only fully decomposed after 7 h. The ligands (**L**_iq_ and **L**_q_) and cages (**C**_iq_ and **C**_q_) were assessed for their cytotoxic properties against two cancerous cell lines (A549 lung cancer cells and MDA-MB-231 breast cancer cells) and one non-cancerous cell line (HDFa skin cells). It was found that **L**_q_ and **C**_q_ were both reasonably cytotoxic against A549, while **C**_iq_ was slightly less active. The higher observed cytotoxicity of **C**_q_ relative to the other [Pd_2_(**L**)_4_]^4+^ architectures was presumed to be due the favorable combination of high hydrophobicity and the kinetic robustness against biological nucleophiles. However, none of the new molecules showed any selectivity for cancer cells, they were all found to have similar cytotoxicity against HDFa skin cells. A range of [Pd_2_(**L**)_4_]^4+^ cage systems have now been shown to be cytotoxic. However, in order to advance this class of MSA as anticancer agents more in depth mode of action/mechanistic studies on the origins of the cytotoxic activity are required. Studies to this effect are now underway.

## Author contributions

RV and JC conceived the idea, analyzed the data and wrote the manuscript. RV and LG conducted the synthesis. RV and DP conducted stability studies. RV, DP, and JJ conducted cytotoxicity studies. GG oversaw the cytotoxicity studies and analyzed the data. All authors provided feedback on the manuscript drafts and approved the submission.

### Conflict of interest statement

The authors declare that the research was conducted in the absence of any commercial or financial relationships that could be construed as a potential conflict of interest.
