# Supplementary material for: Anticancer Activity and Cisplatin Binding Ability of Bis-Quinoline and Bis-Isoquinoline Derived [Pd2L4]4+ Metallosupramolecular Cages
Source: Front Chem. 2018 Nov 22;6:563. doi: 10.3389/fchem.2018.00563 (PMC6262750; doi:10.3389/fchem.2018.00563)
Supplement: Supplementary file 1 [file Data_Sheet_1.docx]

Supplementary Material

Anticancer activity and cisplatin binding ability of *bis*-quinoline and *bis*-isoquinoline derived [Pd_2_L_4_]^4+^ metallosupramolecular cages

Roan A.S. Vasdev,* Lachlan F. Gaudin, Dan Preston, Jackmil P. Jogy, Gregory I. Giles, and James D. Crowley*

*** Correspondence:** Roan Vasdev: [roan.vasdev@otago.ac.nz](mailto:roan.vasdev@otago.ac.nz) and James Crowley: jcrowley@chemistry.otago.ac.nz

**Contents**

[1 Experimental Procedures 2](#_Toc525295558)

[1.1 General 2](#_Toc525295559)

[1.2 Synthesis of L_q_ 4](#_Toc525295560)

[1.3 Synthesis of L_iq_ 6](#_Toc525295561)

[1.4 Synthesis of C_q_ 8](#_Toc525295562)

[1.5 Synthesis of C_iq_ 10](#_Toc525295563)

[2 NMR Data 12](#_Toc525295564)

[2.1 ^1^H DOSY NMR 12](#_Toc525295565)

[3 Time Course ^1^H NMR Stability Studies 15](#_Toc525295566)

[3.1 ^1^H NMR Time Course Stackplots 15](#_Toc525295567)

[4 Cytotoxicity Studies 16](#_Toc525295568)

[4.1 Methods 16](#_Toc525295569)

[5 Calculations 20](#_Toc525295570)

[6 X-ray Data 21](#_Toc525295571)

[6.1 C_q_ 21](#_Toc525295572)

[6.2 Crystallographic Data 23](#_Toc525295573)

[7 References 25](#_Toc525295574)

# Experimental Procedures

## General

Unless otherwise stated, all reagents were purchased from commercial sources and used without further purification. **2** (Sakamoto et al., 1983), **3** (Sakamoto et al., 1983), **5** (Dutta et al., 2014) and **6** (Sakamoto et al., 1983) were synthesised according to the corresponding literature procedures. Solvents were laboratory reagent grade. ^1^H and ^13^C NMR spectra were recorded on either a 400 MHz Varian 400 MR or Varian 500 MHz VNMRS spectrometer. Chemical shifts are reported in parts per million and referenced to residual solvent peaks (CDCl_3_: ^1^H δ 7.26 ppm, ^13^C δ 77.16 ppm; CD_3_CN: ^1^H δ 1.94, ^13^C δ 1.32, 118.26 ppm, *d*_6_‑DMSO: ^1^H δ 2.50 ppm; ^13^C δ 39.52 ppm). Coupling constants (J) are reported in Hertz (Hz). Standard abbreviations indicating multiplicity were used as follows: m = multiplet, q = quartet, t = triplet, dt = double triplet, d = doublet, dd = double doublet, s = singlet. IR spectra were recorded on a Bruker ALPHA FT-IR spectrometer with an attached ALPHA-P measurement module. Microanalyses were performed at the Campbell Microanalytical Laboratory at the University of Otago. Electrospray mass spectra (ESMS) were collected on a Bruker micro-TOF-Q spectrometer; spectra for cages **C_q_** and **C_iq_** were obtained under cold-spray conditions at -10 °C.

Scheme S1: Ligand synthesis scheme. Conditions: (i) ethynyltrimethylsilane, [Pd(PPh_3_)_3_Cl_2_], CuI, (*i*Pr)_2_NH, 80 °C; (ii) Na_2_CO_3_, CH_3_OH, RT; (iii) 2,6-dibromopyridine, [Pd(PPh_3_)_3_Cl_2_], CuI, (*i*Pr)_2_NH, 80 °C; (iv) [Pd(CH_3_CN)_4_](BF_4_)_2_, CH_3_CN, 65 °C (for L_q_ 🡪 C_q_), RT (for L_iq_ 🡪 C_iq_).

## Synthesis of L_q_

THF (1 mL) and (*i*Pr)_2_NH (4 mL) were degassed in a glass tube before 2,6-dibromopyridine (287 mg, 1.212 mmol), 3-ethynylquinoline (**3**) (390 mg, 2.55 mmol), CuI (23 mg, 0.121 mmol) and [Pd(PPh_3_)_2_Cl_2_] (22 mg, 0.030 mmol) were added. The tube was sealed and heated at 80 °C for 14 hours. After cooling to RT, the reaction mixture taken up in CHCl_3_ and was washed with an aqueous EDTA/NH_4_OH solution (0.1 M, 20 mL). The organic layer was washed with water (2 x 20 mL) and brine (20 mL) before drying over Na_2_SO_4_. The solvent was removed under vacuum and the crude product was purified through column chromatography (silica gel CH_2_Cl_2_ to 1:4 acetone/CH_2_Cl_2_) Yield: 400 mg, 1.05 mmol, 86%. ^1^H NMR (400 MHz, **CD_3_CN**, 298 K) *δ*: 9.04 (d, *J* = 1.9 Hz, 2H, H_a_), 8.55 (s, 2H, H_f_), 8.08 (d, *J* = 8.5 Hz, 2H, H_b_), 7.95 (d, *J* = 8.2 Hz, 2H, H_e_), 7.88 (t, *J* = 7.9 Hz, 1H, H_h_), 7.81 (t, *J* = 7.7 Hz, 2H, H_d_), 7.70 – 7.62 (m, 4H, H_g,c_). ^13^C NMR (125 MHz, **CD_3_CN**, 298 K) *δ*: 152.8, 148.2, 144.1, 140.3, 138.5, 137.7, 131.8, 130.2, 129.0, 128.6, 128.1, 128.0, 91.7, 87.2. HRESI-MS (MeOH): *m/z* = 382.1324 [**M** + H]^+^ (calc. for C_27_H_16_N_3_, 382.1339); 763.2560 [2**M** + H]^+^ (calc. for C_54_H_33_N_6_, 763.2610); 785.2377 [2**M** + Na]^+^ (calc. for C_54_H_32_N_6_Na, 785.2430); Anal. Calc. for C_27_H_15_N_3_·0.4H_2_O C, 83.44; H, 4.10; N, 10.81%. Found C, 83.53; H, 4.08; N, 10.92%.


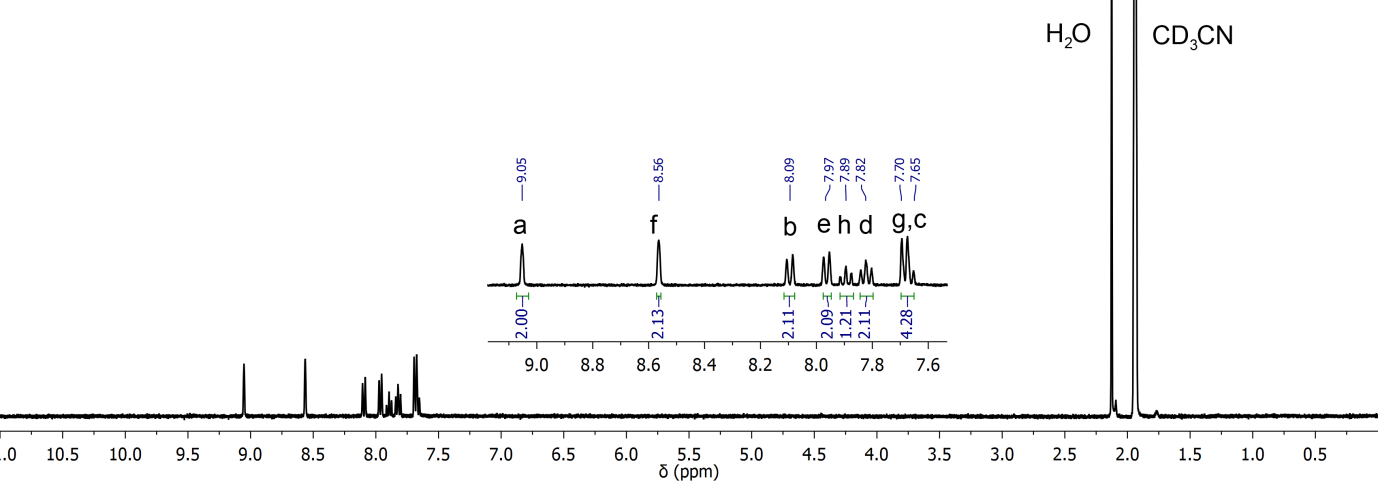


Figure S 1: ^1^H NMR (400 MHz, CD_3_CN, 298 K) of **L_q_**.


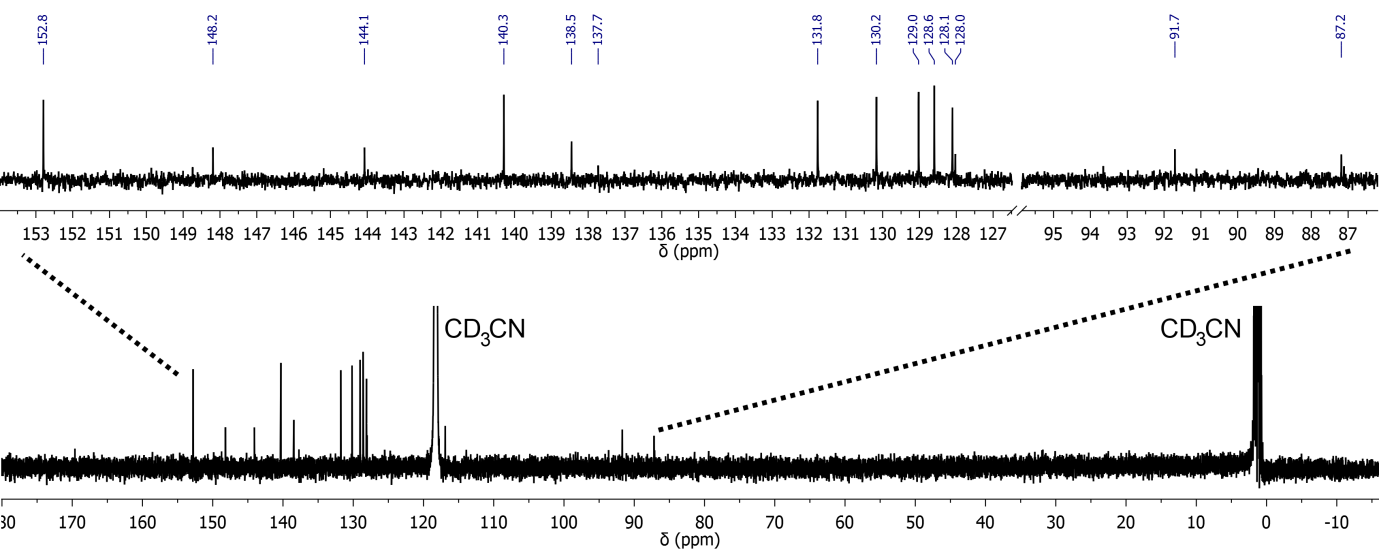


Figure S 2: ^13^C NMR (125 MHz, CD_3_CN, 298 K) of **L_q_**.

## Synthesis of L_iq_

THF (1 mL) and (*i*Pr)_2_NH (4 mL) were degassed in a glass tube before 2,6-dibromopyridine (267 mg, 1.127 mmol), 4-ethynylisoquinoline (**6**) (371 mg, 2.15 mmol), CuI (22 mg, 0.113 mmol) and [Pd(PPh_3_)_2_Cl_2_] (20 mg, 0.028 mmol) were added. The tube was sealed and heated at 80 °C for 14 hours. After cooling to RT, the reaction mixture taken up in CHCl_3_ and was washed with an aqueous EDTA/NH_4_OH solution (0.1 M, 20 mL). The organic layer was washed with water (2 x 20 mL) and brine (20 mL) before drying over Na_2_SO_4_. The solvent was removed under vacuum and the crude product was purified through column chromatography (silica gel CH_2_Cl_2_ to 1:4 acetone/CH_2_Cl_2_). Yield: 335 mg, 0.879 mmol, 78%. ^1^H NMR (400 MHz, **CDCl_3_**, 298 K) *δ*: 9.32 (d, *J* = 0.8 Hz, 2H, H_b_), 8.85 (s, 2H, H_a_), 8.53 – 8.48 (m, 2H, H_c_), 8.16 – 8.10 (m, 2H ,H_f_), 7.97 (ddd, *J* = 8.4, 7.0, 1.2 Hz, 2H, H_e_), 7.86 (dd, *J* = 8.3, 7.3 Hz, 1H, H_e_), 7.83 – 7.78 (m, 2H, H_d_), 7.71 (dd, *J* = 7.8, 0.5 Hz, 2H, H_g_). ^13^C NMR (125 MHz, **CD_3_CN**, 298 K) *δ*: 154.2, 148.1, 144.2, 138.5, 136.2, 132.9, 132.7, 132.6, 129.4, 129.3, 128.3, 125.5, 89.7, 85.1. HRESI-MS (**CH_3_OH**): *m/z* = 382.1320 [**M** + H]^+^ (calc. for C_27_H_16_N_3_, 382.1339); 404.1132 [**M** + Na]^+^ (calc. for C_27_H_15_N_3_Na, 404.1158); Anal. Calc. for C_27_H_15_N_3_·0.50CH_3_CN C, 83.74; H, 4.13; N, 12.13%. Found C, 83.35; H, 4.10; N, 11.79%.


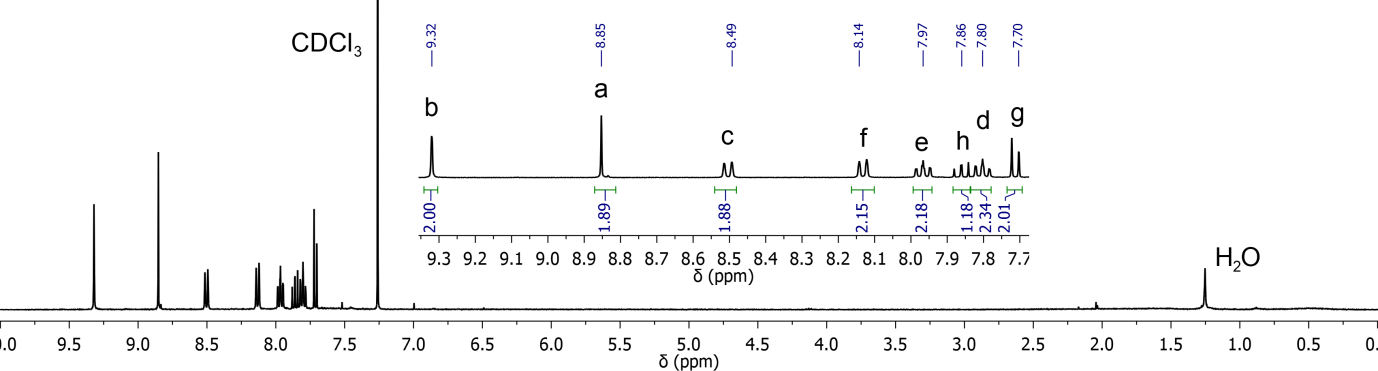


Figure S 3: ^1^H NMR (400 MHz, CDCl_3_, 298 K) of **L_iq_**.


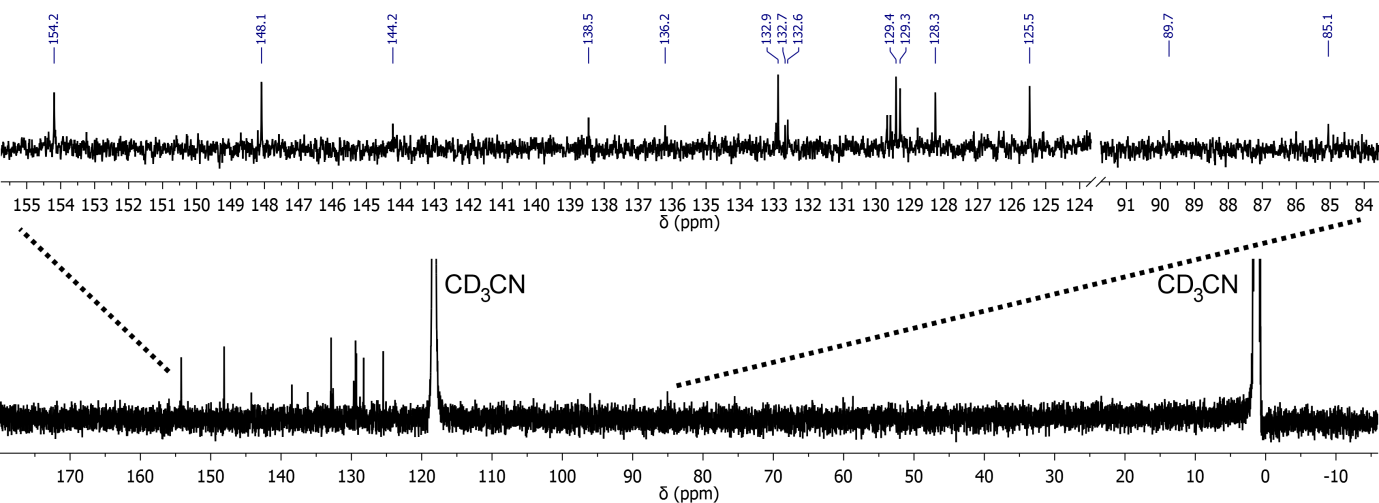


Figure S 4: ^13^C NMR (125 MHz, CD_3_CN, 298 K) of **L_iq_**.

## Synthesis of C_q_

**L_q_** (50 mg, 0.131 mmol) and [Pd(CH_3_CN)_4_](BF_4_)_2_ (29 mg, 0.066 mmol) were dissolved in MeCN (10 mL) and heated at 65 °C for 7 hours. The resulting solution was cooled to RT and precipitated via addition of diethyl ether. The tan solid was collected through filtration and washed with diethyl ether to give the product. Yield: 63 mg, 0.030 mmol 92%. ^1^H NMR (400 MHz, **CD_3_CN**, 298 K) *δ*: 9.73 (s, 8H, H_a_), 9.61 (d, *J* = 8.8 Hz, 8H, H_b_), 8.75 (s, 8H, H_f_), 7.80 – 7.93 (m, 16H, H_e,c_), 7.85 (t, *J* = 7.9 Hz, 4H, H_h_), 7.79 (t, *J* = 7.6 Hz, 8H, H_d_), 7.66 (d, *J* = 7.8 Hz, 8H, H_g_). ^13^C NMR (125 MHz, **CD_3_CN**, 298 K) *δ*: 156.9, 146.5, 145.6, 143.5, 142.3, 135.4, 131.0, 130.9, 129.9, 129.6, 128.0, 119.4, 93.8, 84.4. ^19^F NMR (470 MHz, **CD_3_CN**, 298 K) *δ*: -155.85. HRESI-MS (**CH_3_CN**): *m/z* = 434.5856 [**C_q_** – 4BF_4_]^4+^ (calc. for (C_27_H_15_N_3_)_4_Pd_2_, 434.5789); 585.7749 [**C_q_** – 4BF_4_ + F]^3+^ (calc. for (C_27_H_15_N_3_)_4_Pd_2_·F, 585.7716); 608.4439 [**C_q_** – 3BF_4_]^3+^ (calc. for (C_27_H_15_N_3_)_4_Pd_2_·BF_4_, 608.4400); 922.1596 [**C_q_** – 3BF_4_ + F]^2+^ (calc. for (C_27_H_15_N_3_)_4_Pd_2_·BF_4_·F, 922.1595); 956.1634 [**C_q_** – 2BF_4_]^2+^ (calc. for (C_27_H_15_N_3_)_4_Pd_2_·2BF_4_, 956.1621).


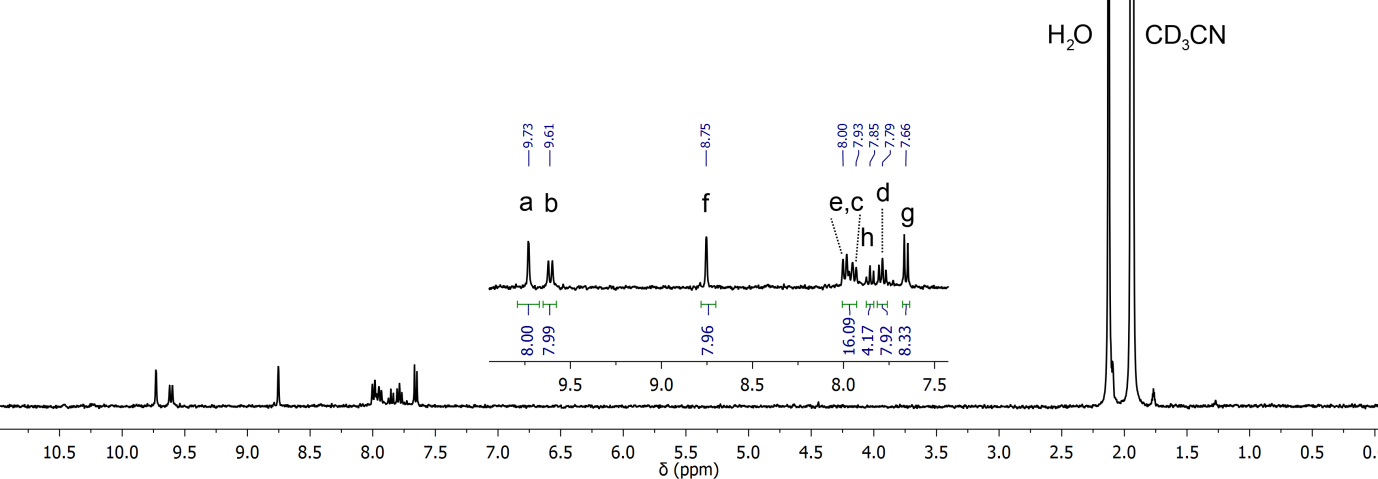


Figure S 5: ^1^H NMR (400 MHz, CD_3_CN, 298 K) of **C_q_**.


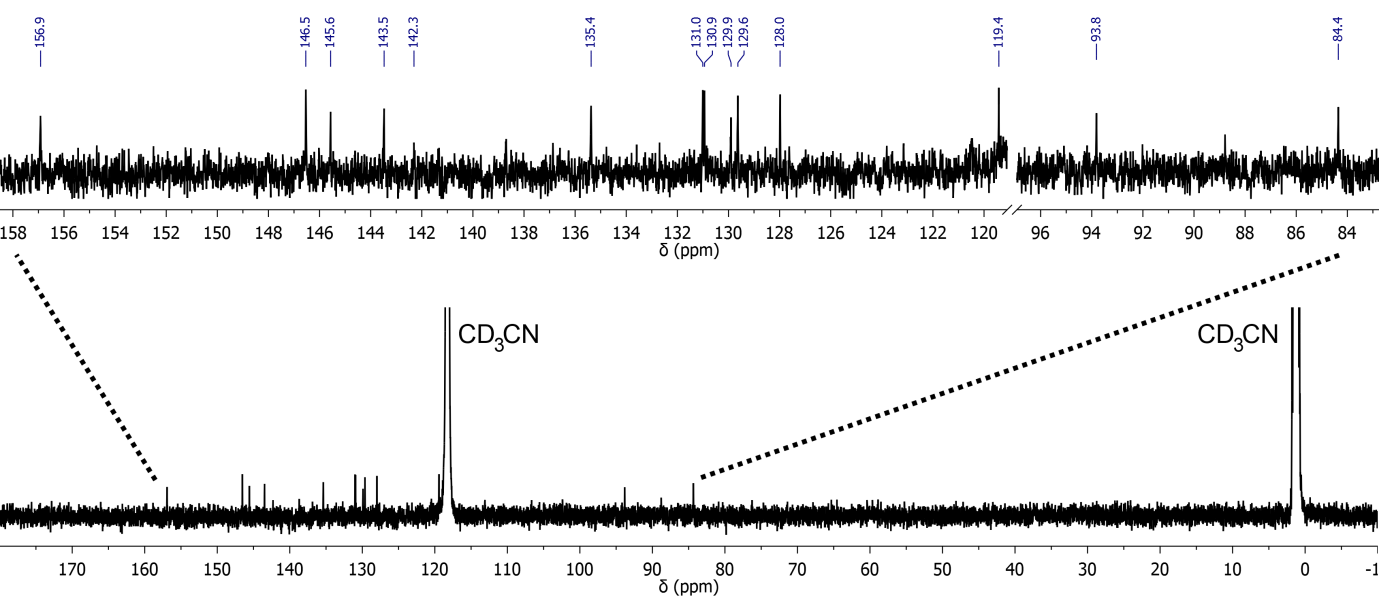


Figure S 6: ^13^C NMR (125 MHz, CD_3_CN, 298 K) of **C_q_**.


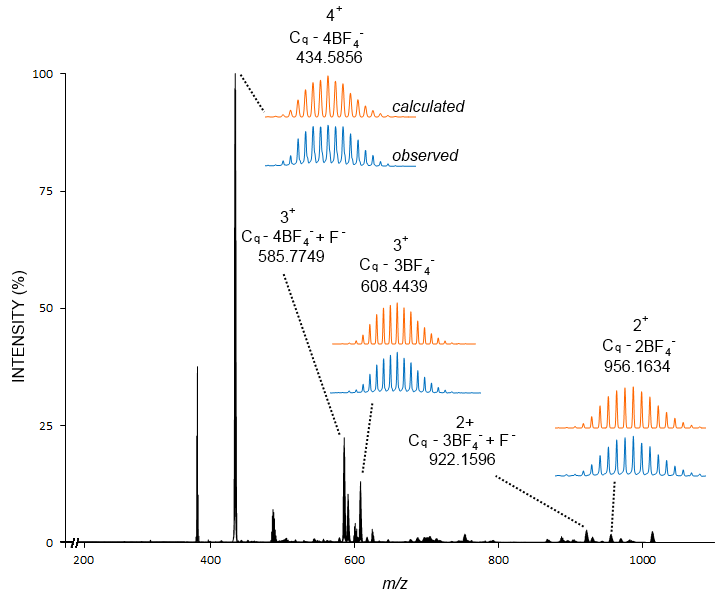


Figure S 7: Partial HR-ESMS (CH_3_CN, 263 K) spectrum of **C_q_**.

## Synthesis of C_iq_

**L_iq_** (100 mg, 0.262 mmol) and [Pd(CH_3_CN)_4_](BF_4_)_2_ (58 mg, 0.131 mmol) were added to MeCN (15 mL) and stirred at RT for 1 hour. To the resulting mixture was added diethyl ether, and the ensuing white precipitate was collected through filtration and washed with diethyl ether giving the product as a tan solid. Yield: 120 mg, 0.058 mmol, 88%. ^1^H NMR (500 MHz, **CD_3_CN**, 298 K) *δ*: 9.99 (s, 8H, H_b_), 9.40 (s, 8H, H_a_), 8.45 (dd, *J* = 8.2, 1.0 Hz, 8H, H_c_), 8.34 (dd, *J* = 8.5, 1.1 Hz, 8H, H_f_), 8.07 (ddd, *J* = 8.2, 6.9, 1.2 Hz, 8H, H_d_), 7.96 – 7.86 (m, 20H, H_e,g,h_). ^13^C NMR (125 MHz, **CD_3_CN**, 298 K) *δ*: 157.2, 145.8, 143.5, 138.6, 137.0, 136.5, 131.6, 130.6, 129.8, 129.7, 125.8, 120.1, 98.6, 82.3. ^19^F NMR (470 MHz, **CD_3_CN**, 298 K) *δ*: -155.52. HRESI-MS (**CH_3_CN**): *m/z* = 434.5832 [**C_iq_**– 4BF_4_]^4+^ (calc. for (C_27_H_15_N_3_)_4_Pd_2_, 434.5789); 585.7744 [**C_iq_** – 4BF_4_ + F]^3+^ (calc. for (C_27_H_15_N_3_)_4_Pd_2_·F, 585.7716); 608.4424 [**C_iq_** – 3BF_4_]^3+^ (calc. for (C_27_H_15_N_3_)_4_Pd_2_·BF_4_, 608.4400); 956.1610 [**C_iq_** – 2BF_4_]^2+^ (calc. for (C_27_H_15_N_3_)_4_Pd_2_·2BF_4_, 956.1622).


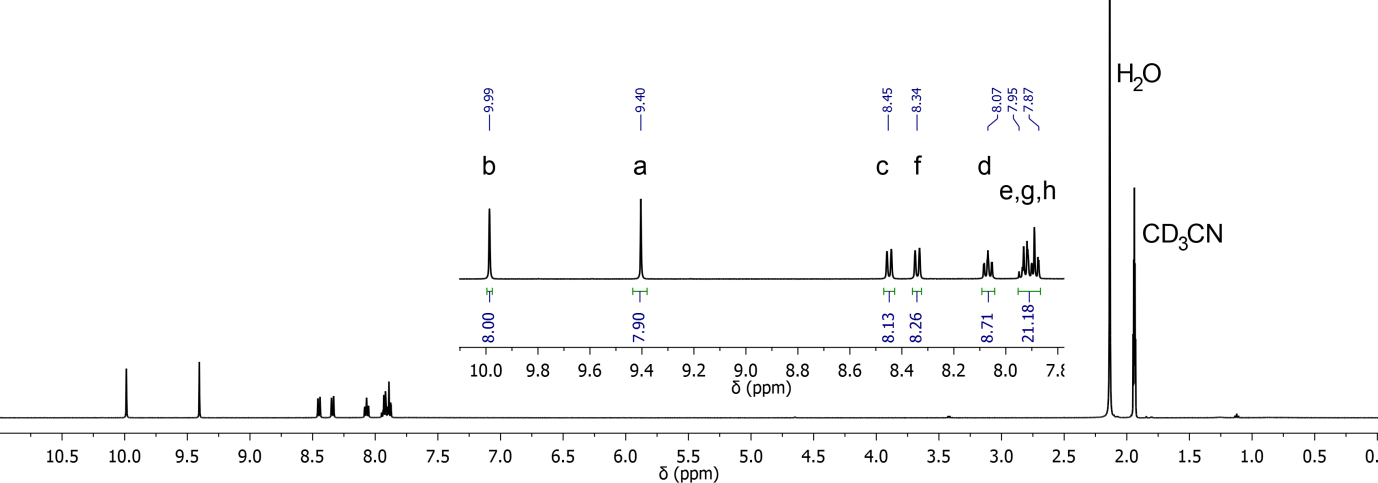


Figure S 8: ^1^H NMR (500 MHz, CD_3_CN, 298 K) of **C_iq_**.


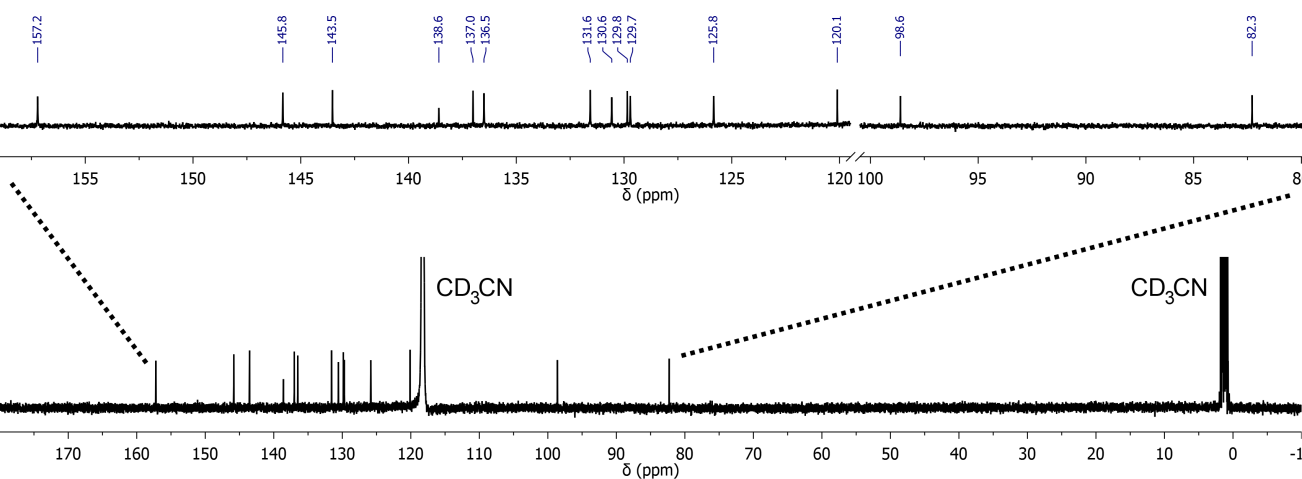


Figure S 9: ^13^C NMR (125 MHz, CD_3_CN, 298 K) of **C_iq_**.


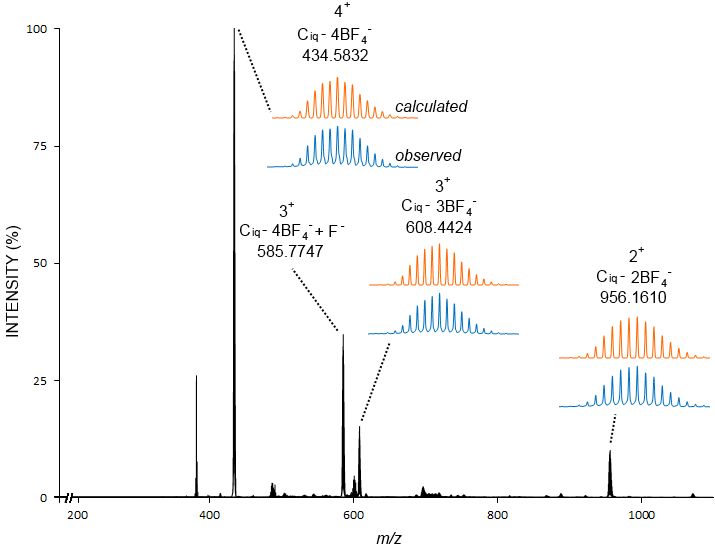


Figure S 10: Partial HR-ESMS (CH_3_CN, 263 K) spectrum of **C_iq_**.

# NMR Data

## ^1^H DOSY NMR


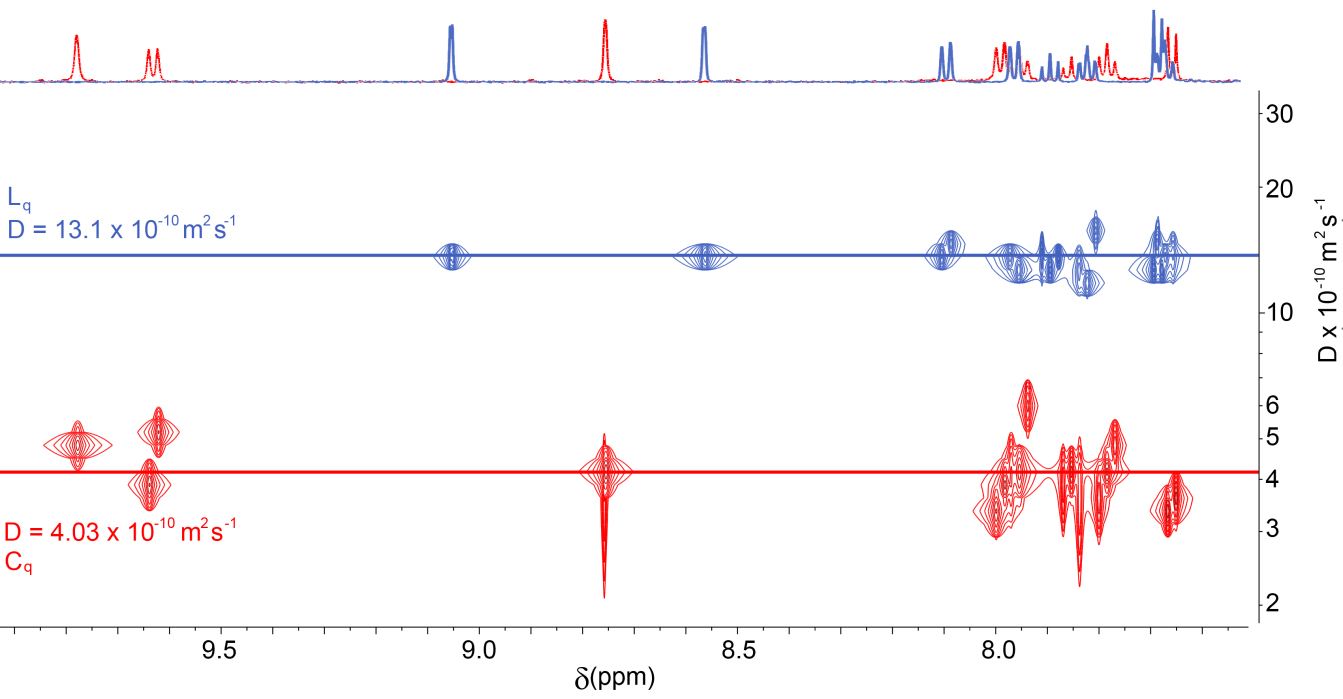


Figure S 11: Partial ^1^H DOSY NMR (500 MHz, CD_3_CN, 298 K) spectra of **L_q_** and **C_q_**.


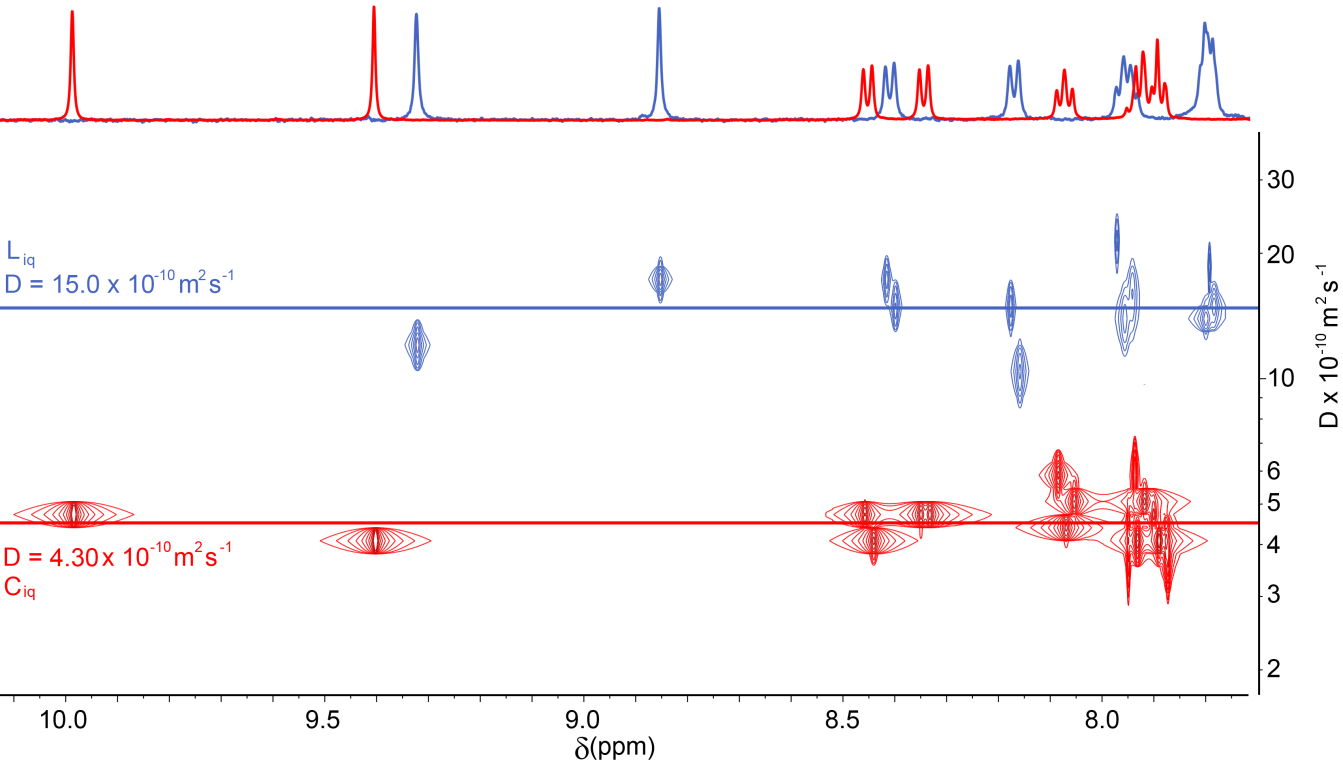


Figure S 12: Partial ^1^H DOSY NMR (500 MHz, CD_3_CN, 298 K) spectra of **L_iq_** and **C_iq_**.

Table S 1: ^1^H DOSY NMR data – derived diffusion coefficients (*D*) (500 MHz, CD_3_CN, 298 K).

|  | **M_W_ (g mol^-1^)** | **log(M_W_)** | **D (x 10^-10^ m^2^ s^-1^)** | **log(D)** |
| --- | --- | --- | --- | --- |
| **L_q_** | 381 | 2.581 | 13.1 | 1.119 |
| **L_iq_** | 381 | 2.581 | 15.0 | 1.175 |
| **C_q_** | 2086 | 3.319 | 4.07 | 0.610 |
| **C_iq_** | 2086 | 3.319 | 4.30 | 0.634 |
| **L_tripy_** | 282 | 2.451 | 15.9 | 1.202 |
| **C_tripy_** | 1693 | 3.229 | 5.87 | 0.769 |
| **L_hexox_** | 482 | 2.683 | 11.5 | 1.062 |
| **C_hexox_** | 2487 | 3.396 | 3.65 | 0.563 |
| **L_peg_** | 518 | 2.714 | 10.6 | 1.024 |
| **C_peg_** | 2626 | 3.419 | 5.07 | 0.705 |


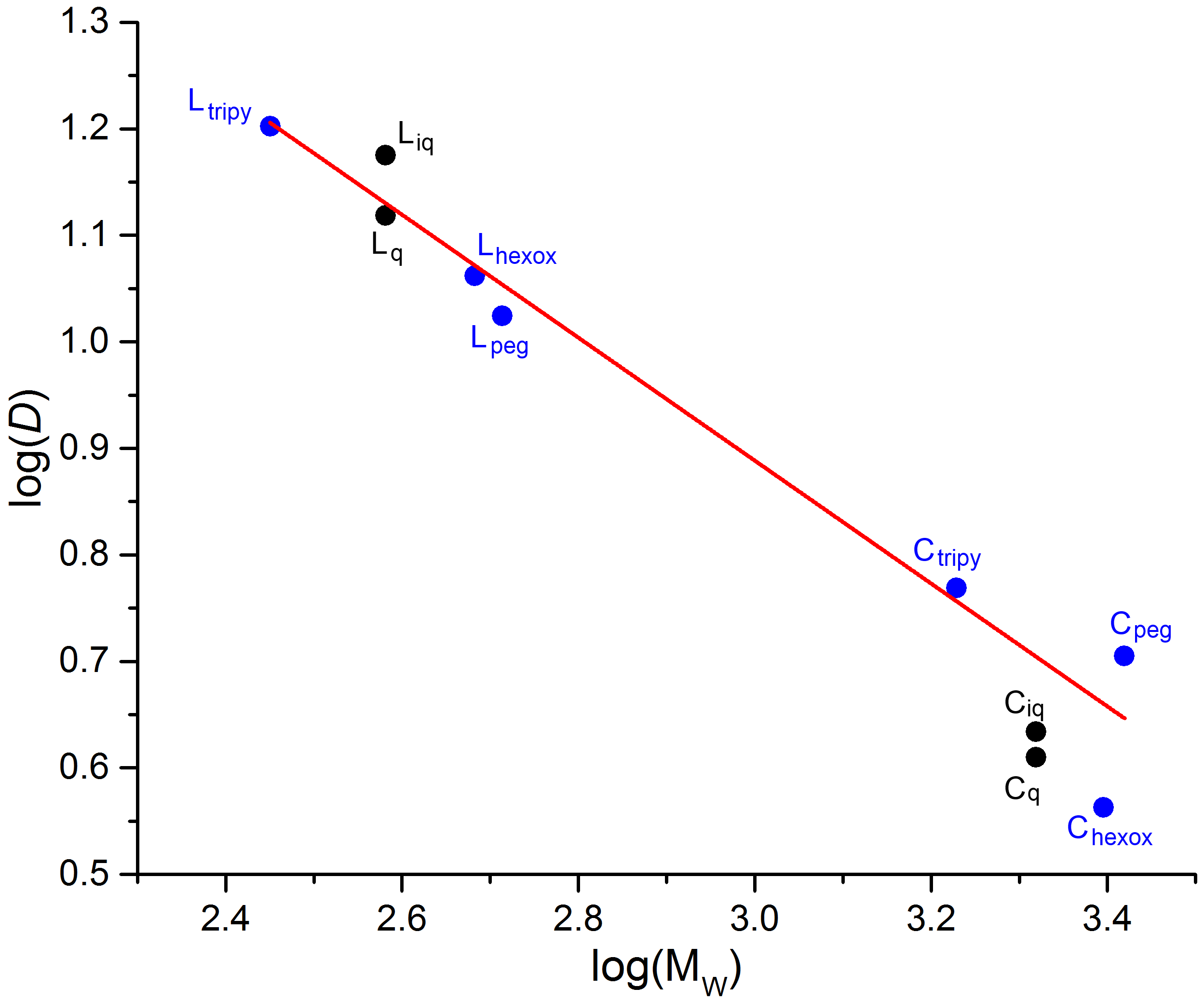


Figure S 13: Plot of log(*D*) against log(M_W_) (500 MHz, CD_3_CN, 298 K), diffusion coefficients: ×10^–10^ m^2^ s^−1^ and molecular weights: g mol^−1^. **L_tripy_** and **C_tripy_** refer to the original tripyridyl ligand and cage for reference. The linear fit to the data gives the equation: log(*D*) = 2.62 – 0.577log(M_W_) with an R^2^ of 0.906.

# Time Course ^1^H NMR Stability Studies

All time course studies were carried out on a Varian 500 MHz VNMRS spectrometer at 298 K (*d*_6_-DMSO). The concentration of the [Pd_2_(**L**)_4_]^4+^ architectures was in all cases 2 mM. Testing was carried out against tetrabutylammonium chloride (8 eq.). A reference sample of each [Pd_2_(**L**)_4_]^4+^ cage at the correct concentration without nucleophile was used for time zero.

## ^1^H NMR Time Course Stackplots

The only ^1^H NMR peaks pertaining to the progressing reaction against chloride in the aromatic region belonged to cage. In the case of **C_q_**, introduction of tetrabutylammonium chloride had little to no effect for the first hour of the study.


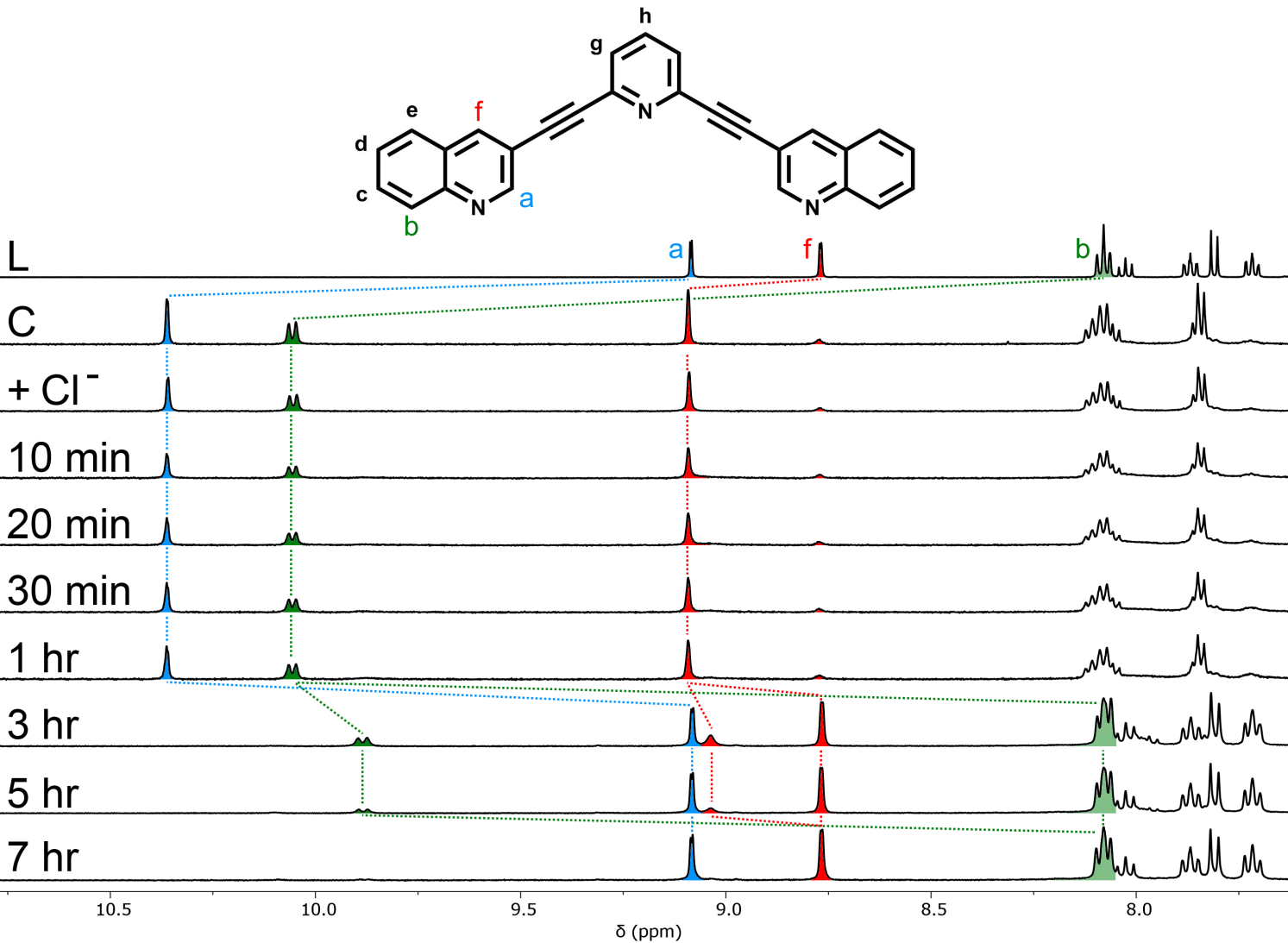


Figure S 14: Partial ^1^H NMR stacked spectra (500 MHz, *d*_6_-DMSO, 298 K) of C_q_ upon addition of 8 eq. Cl^-^.

For the **C_iq_** system, instantanoues degradation occurred, creating multiple species.


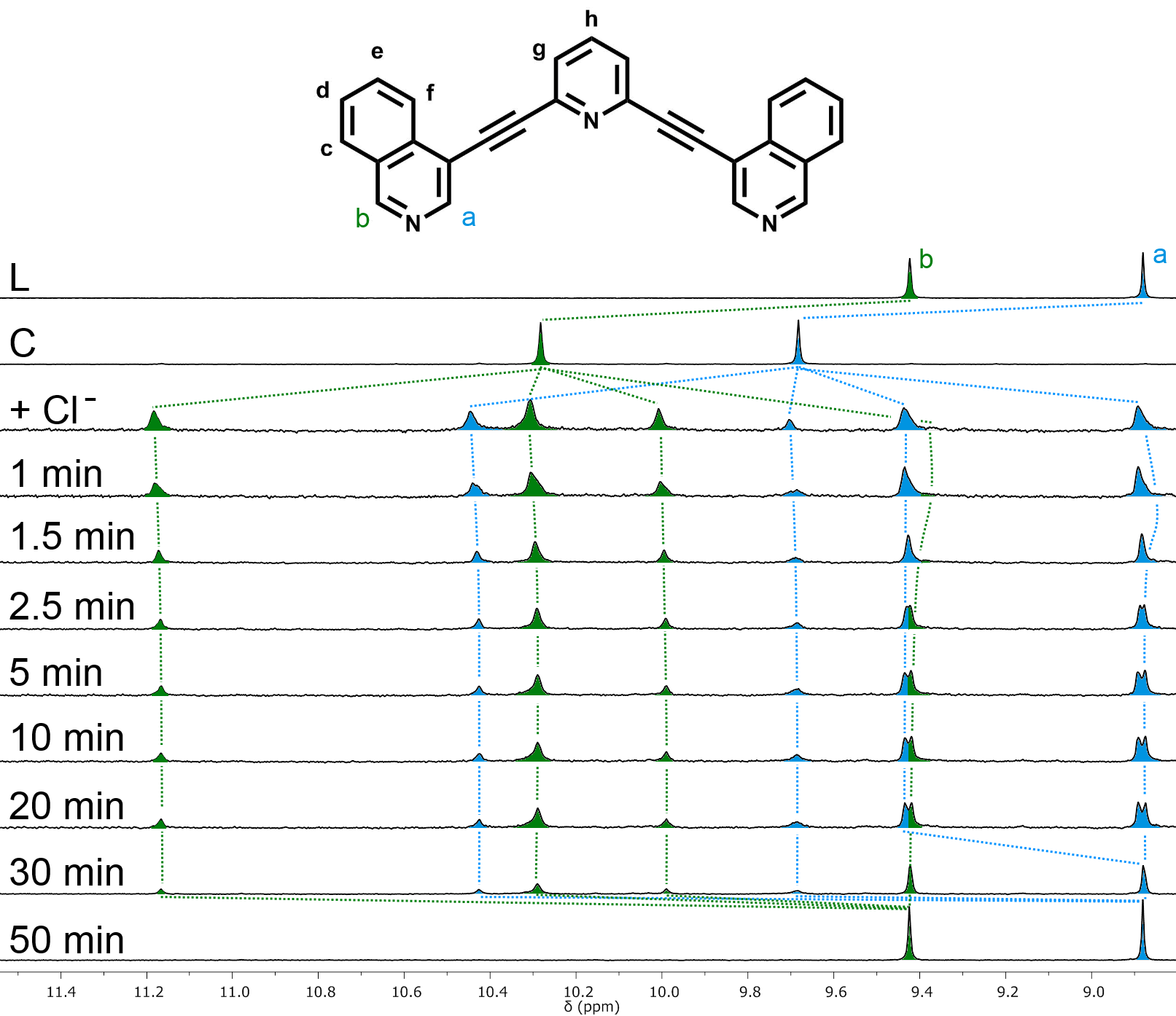


Figure S 15: Partial ^1^H NMR stacked spectra (500 MHz, *d*_6_-DMSO, 298 K) of **C_iq_** upon addition of 8 eq. Cl^-^.

# Cytotoxicity Studies

## Methods

**Reagents:** MTT (3-[4,5-dimethylthiazol-2-yl]-2,5-diphenyltetrazolium bromide) and cell culture reagents were purchased from Invitrogen (Auckland, NZ). All other chemicals were obtained from Sigma-Aldrich (Auckland, NZ) unless stated otherwise.

**Cell Culture:** All cell lines were purchased from the American Type Culture Collection (ATCC, Manassas, VA, USA) and maintained in Dulbecco’s Modified Eagles Medium (DMEM) enriched with 2% FBS and 1% antibiotic. All cells were cultured at 37 °C in a humidified atmosphere with 5% CO_2_ levels.

**Cytotoxicity Evaluation**: Cell viability was assessed using the MTT assay.(Mosmann, 1983) 96 well plates were seeded with 5,000 cells per well, and the cells left to adhere for 24 hours prior to treatment. Cells were then exposed to compounds solubilised in DMSO. To control for the effects of DMSO, all cell culture medium had a constant DMSO concentration of 0.5% (v/v). Following compound administration, cells were washed with PBS and MTT (0.33 mg mL^-1^ in DMEM) added for 3 hours. After MTT incubation, the medium was aspirated and the residual crystals dissolved in DMSO. Cell number was then calculated at λ = 550 nm.

Table S 2: Half-maximal inhibitory concentrations (IC_50_) of ligands **L_q_** and **L_iq_**, and cages **C_q_** and **C_iq_** architectures at 24 h. ^a^Solubility limited the range of concentrations available to below 1 μM.

| **Compound** | **IC_50_ (μM)** | | |
| --- | --- | --- | --- |
|  | **MDA-MB-231** | **A549** | **HDFa** |
| **L_q_** | > 1^a^ | 0.5 ± 0.1 | > 1^a^ |
| **C_q_** | 1.7 ± 0.1 | 0.5 ± 0.1 | 2.6 ± 0.4 |
| **L_iq_** | > 1^a^ | > 1^a^ | > 1^a^ |
| **C_iq_** | 4.0 ± 0.3 | 7.4 ± 1.0 | 3.0 ± 0.4 |


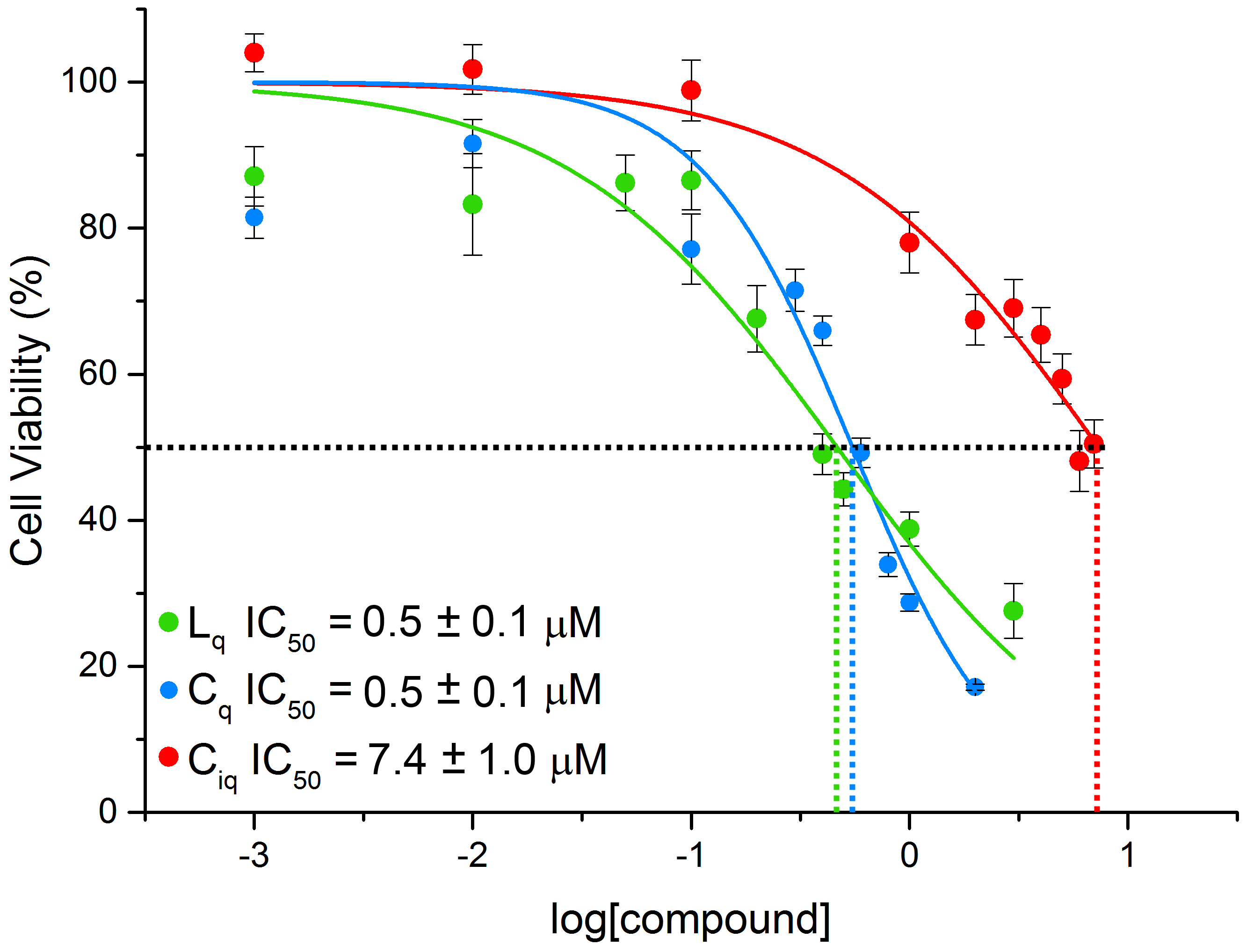


Figure S 16: Viability of A549 cells against L_q,_ C_q_ and C_iq_ architectures at 24 h.


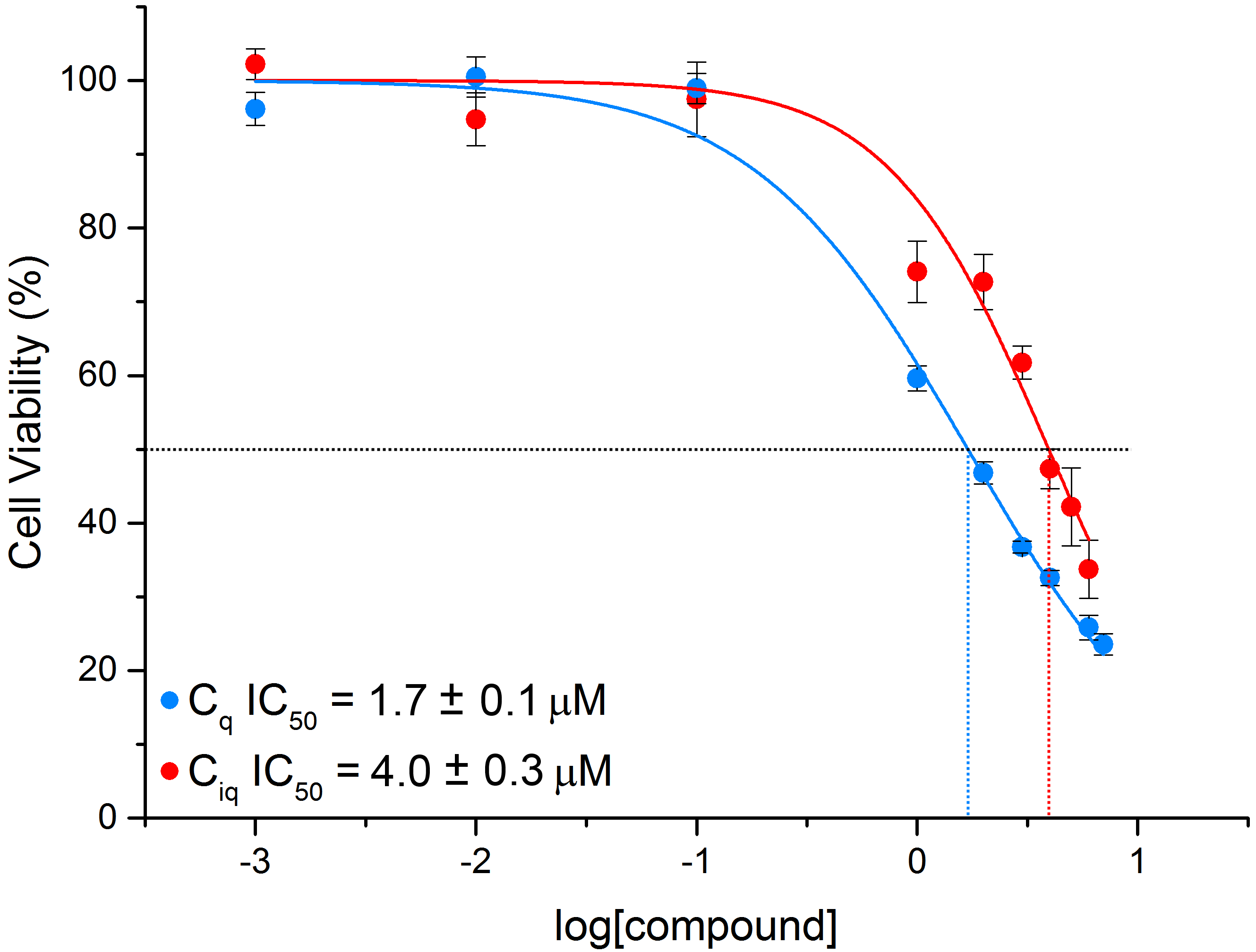


Figure S 17: Viability of MDA-MB-231 cells against C_q_ and C_iq_ architectures at 24 h.


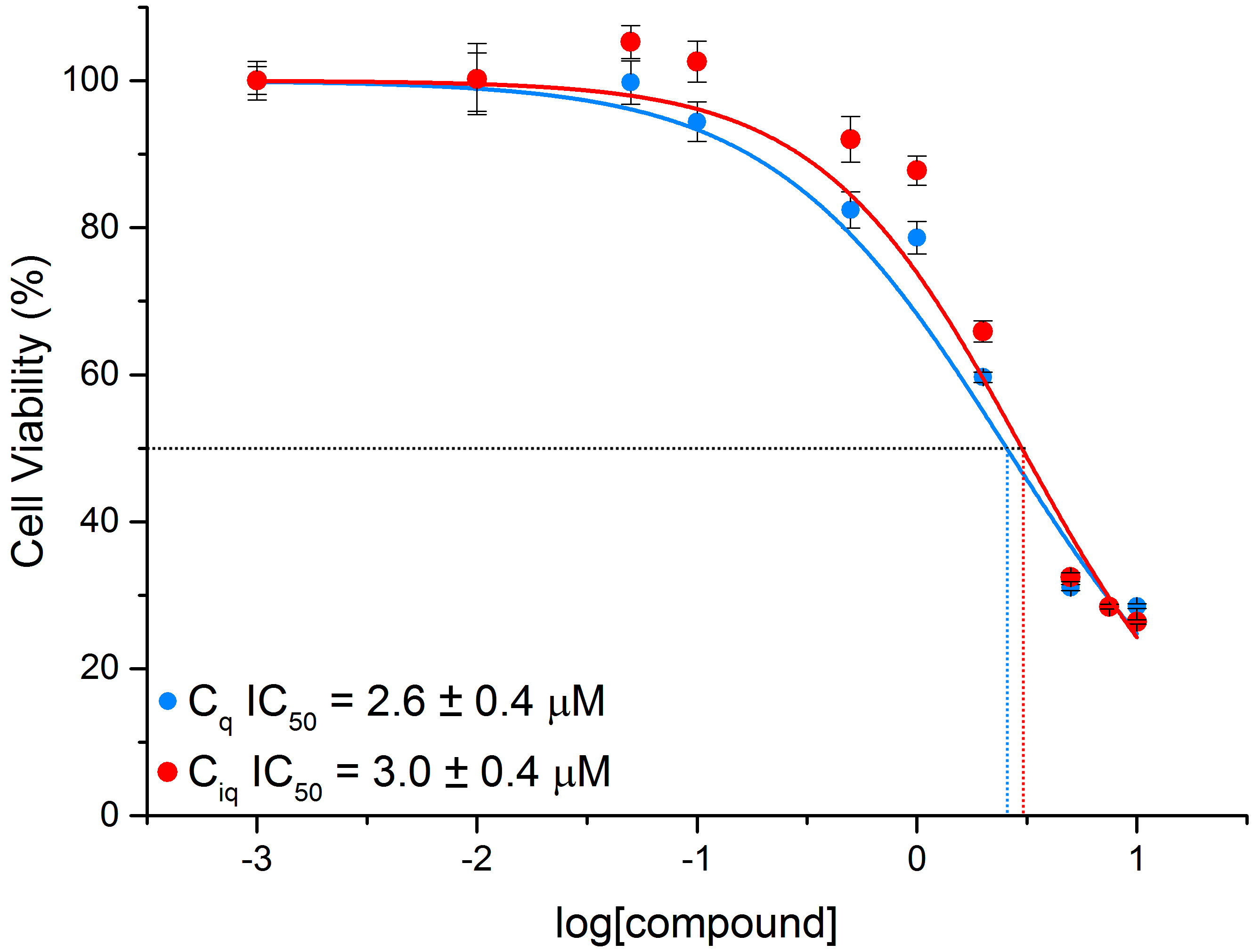


Figure S 18: Viability of HDFa cells against C_q_ and C_iq_ architectures at 24 h.

# Calculations

All DFT calculations were performed using the ORCA program version 4.0 (Neese, 2012). All cages were fully optimized using the BP86 (Perdew, 1986; Perdew and Yue, 1986; Becke, 1988) functional with a def2-SVP basis set (Schaefer et al., 1992). The resolution of identity approximation (Neese, 2003) was also used in the BP86 calculations, with a def2-SVP/J auxiliary basis set (Eichkorn et al., 1995a; b; Eichkorn et al., 1997). Calculations were performed in a polarizable continuum solvent using both the COSMO (Klamt and Schueuermann, 1993; Klamt, 1995; Klamt and Jonas, 1996) and SMD (Marenich et al., 2009) solvation models with acetonitrile (ε=35.6880). SCF iterations were considered converged when the energy change was less than 1×10^-8^ a.u. The geometry was considered optimized when the following tolerances were met: Gradient = 3×10^-4^ a.u., RMS gradient = 1×10^-4^ a.u., maximum gradient = 3×10^-4^ a.u., RMS displacement = 2×10^-3^ a.u., maximum displacement = 4×10^-3^ a.u.. To reduce numerical error in the DFT integration, more grid points were used for both the angular and radial grids via the keyword “Grid4” for the SCF iterations and “Grid5” for the final energy evaluation.


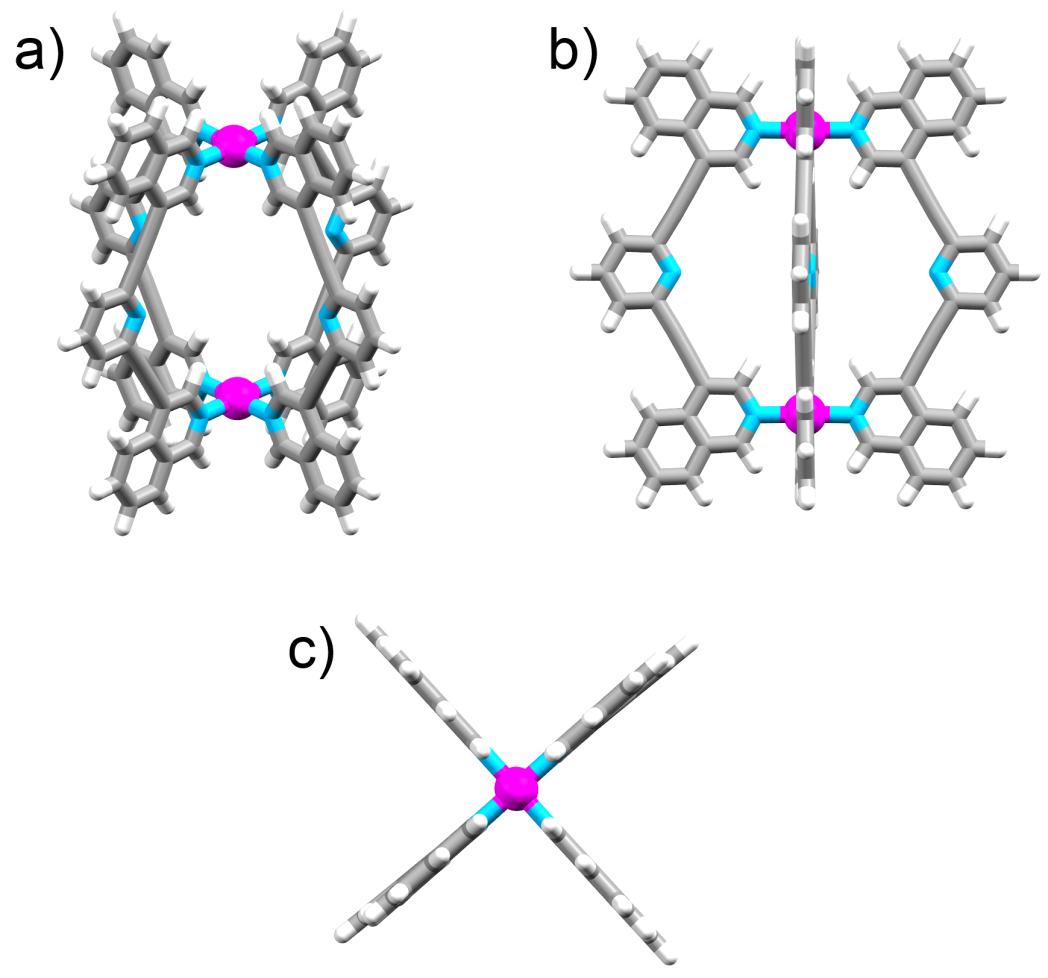


Figure S 19: Optimized geometry (BP86 def2-SVP, MeCN solvent field) of Ciq showing lantern-shape conformation.

# X-ray Data

## C_q_

Evaporation of an acetonitrile solution of **C_q_** gave colourless block crystals of **C_q_**. X-ray data were collected at 89.8 K on an Agilent Technologies Supernova system using Cu Κα radiation with exposures over 1.0°, and data were treated using the CrysAlisPro(2012) software. Using Olex2,(Dolomanov et al., 2009) the structure was solved with the ShelXT(Sheldrick, 2015b) structure solution program using Intrinsic Phasing and refined with the ShelXL(Sheldrick, 2015a) refinement package using Least Squares minimisation. All non-hydrogen atoms were refined anisotropically. Hydrogen atoms attached to carbons were placed in calculated positions and refined using a riding model. The structure was solved in the tetragonal space group P4/mnc and refined to an R_1_ value of 7.21%. The asymmetric unit contains 50% of one **L_q_** ligand, one palladium (25% occupancy), 25% of a BF­_4_ (boron is 25% occupancy and fluorine is full occupancy) and an acetonitrile solvent molecule. (Figure S 20).


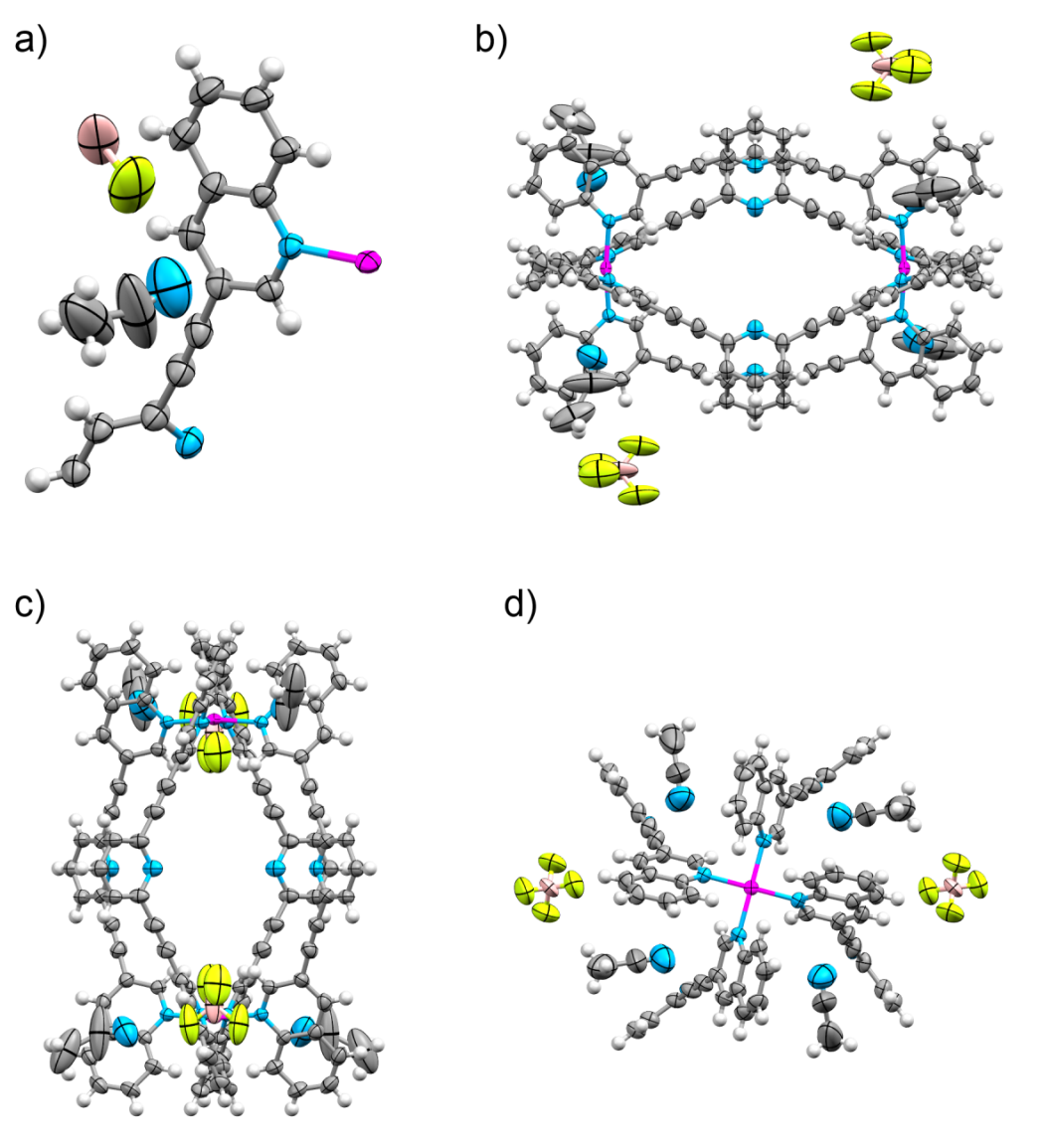


Figure S 20: Mercury ellipsoid plot of a) the asymmetric unit of C_q,_ and b-d) orthogonal views of C_q_. Ellipsoids are shown at the 50% probability level. Colours: carbon grey, nitrogen blue, hydrogen white, palladium magenta, boron salmon, fluorine yellow.

The SQUEEZE routine running within PLATON was employed to resolve the diffuse electron density.

Table S 3: SQUEEZE details for C**_q_**.

| Void | X | Y | Z | Volume | Electrons | Content |
| --- | --- | --- | --- | --- | --- | --- |
| 1 | -0.004 | -0.003 | 0.000 | 823 | 217 | 2 x BF_4_ + diffuse solvent |
| 2 | 0.000 | 0.000 | 0.500 | 59 | 43 | MeCN |
| 3 | -0.116 | 0.498 | 0.500 | 823 | 217 | 2 x BF_4_ + diffuse solvent |
| 4 | 0.500 | 0.500 | 0.000 | 59 | 43 | MeCN |

## Crystallographic Data

| Identification code | C_q_ / LFG005 |
| --- | --- |
| CCDC number | 1863983 |
| Empirical formula | C_124_H_84_B_2_F_8_N_20_Pd_2_ |
| Formula weight | 2240.53 |
| Temperature/K | 89.8 |
| Crystal system | tetragonal |
| Space group | P4/mnc |
| a/Å | 16.9365(2) |
| b/Å | 16.9365(2) |
| c/Å | 22.8144(5) |
| α/° | 90 |
| β/° | 90 |
| γ/° | 90 |
| Volume/Å^3^ | 6544.2(2) |
| Z | 2 |
| ρ_calc_g/cm^3^ | 1.137 |
| μ/mm^‑1^ | 2.724 |
| F(000) | 2284.0 |
| Crystal size/mm^3^ | 0.222 × 0.182 × 0.109 |
| Radiation | Cu Kα (λ = 1.54184) |
| 2Θ range for data collection/° | 7.382 to 149.382 |
| Index ranges | -21 ≤ h ≤ 21, -20 ≤ k ≤ 20, -21 ≤ l ≤ 28 |
| Reflections collected | 35093 |
| Independent reflections | 3419 [R_int_ = 0.0401, R_sigma_ = 0.0164] |
| Data/restraints/parameters | 3419/0/182 |
| Goodness-of-fit on F^2^ | 1.069 |
| Final R indexes [I>=2σ (I)] | R_1_ = 0.0721, wR_2_ = 0.2005 |
| Final R indexes [all data] | R_1_ = 0.0779, wR_2_ = 0.2084 |
| Largest diff. peak/hole / e Å^-3^ | 1.22/-0.99 |

# References

(2012). "CrysAlisPro". (Yarnton, U.K.: Agilent Technologies).

Becke, A.D. (1988). Density-functional exchange-energy approximation with correct asymptotic behavior. Phys. Rev. A: Gen. Phys. 38(6), 3098-3100.

Dolomanov, O.V., Bourhis, L.J., Gildea, R.J., Howard, J.A., and Puschmann, H. (2009). OLEX2: a complete structure solution, refinement and analysis program. J. Appl. Cryst. 42(2), 339-341.

Dutta, U., Maity, S., Kancherla, R., and Maiti, D. (2014). Aerobic Oxynitration of Alkynes with tBuONO and TEMPO. Org. Lett. 16(24), 6302-6305. doi: 10.1021/ol503025n.

Eichkorn, K., Treutler, O., Oehm, H., Haeser, M., and Ahlrichs, R. (1995a). Auxiliary basis sets to approximate Coulomb potentials. Chem. Phys. Lett. 240(4), 283-290. doi: 10.1016/0009-2614(95)00621-A.

Eichkorn, K., Treutler, O., Oehm, H., Haeser, M., and Ahlrichs, R. (1995b). Auxiliary basis sets to approximate Coulomb potentials. [Erratum to document cited in CA123:93649]. Chem. Phys. Lett. 242(6), 652-660. doi: 10.1016/0009-2614(95)00838-U.

Eichkorn, K., Weigend, F., Treutler, O., and Ahlrichs, R. (1997). Auxiliary basis sets for main row atoms and transition metals and their use to approximate Coulomb potentials. Theor. Chem. Acc. 97(1-4), 119-124. doi: 10.1007/s002140050244.

Klamt, A. (1995). Conductor-like Screening Model for Real Solvents: A New Approach to the Quantitative Calculation of Solvation Phenomena. J. Phys. Chem. 99(7), 2224-2235. doi: 10.1021/j100007a062.

Klamt, A., and Jonas, V. (1996). Treatment of the outlying charge in continuum solvation models. J. Chem. Phys. 105(22), 9972-9981. doi: 10.1063/1.472829.

Klamt, A., and Schueuermann, G. (1993). COSMO: a new approach to dielectric screening in solvents with explicit expressions for the screening energy and its gradient. J. Chem. Soc., Perkin Trans. 2 (5), 799-805.

Marenich, A.V., Cramer, C.J., and Truhlar, D.G. (2009). Universal Solvation Model Based on Solute Electron Density and on a Continuum Model of the Solvent Defined by the Bulk Dielectric Constant and Atomic Surface Tensions. J. Phys. Chem. B 113(18), 6378-6396. doi: 10.1021/jp810292n.

Mosmann, T. (1983). Rapid colorimetric assay for cellular growth and survival: application to proliferation and cytotoxicity assays. J Immunol Methods 65(1-2), 55-63.

Neese, F. (2003). An improvement of the resolution of the identity approximation for the formation of the Coulomb matrix. J. Comput. Chem. 24(14), 1740-1747. doi: 10.1002/jcc.10318.

Neese, F. (2012). The ORCA program system. Wiley Interdisciplinary Reviews: Computational Molecular Science 2(1), 73-78. doi: doi:10.1002/wcms.81.

Perdew (1986). Density-functional approximation for the correlation energy of the inhomogeneous electron gas. Phys Rev B Condens Matter 33(12), 8822-8824.

Perdew, and Yue (1986). Accurate and simple density functional for the electronic exchange energy: Generalized gradient approximation. Phys Rev B Condens Matter 33(12), 8800-8802.

Sakamoto, T., Shiraiwa, M., Kondo, Y., and Yamanaka, H. (1983). A facile synthesis of ethynyl-substituted six-membered N-heteroaromatic compounds. Synthesis (4), 312-314. doi: 10.1055/s-1983-30319.

Schaefer, A., Horn, H., and Ahlrichs, R. (1992). Fully optimized contracted Gaussian basis sets for atoms lithium to krypton. J. Chem. Phys. 97(4), 2571-2577. doi: 10.1063/1.463096.

Sheldrick, G.M. (2015a). Crystal structure refinement with SHELXL. Acta Cryst. C71 71(Pt 1), 3-8. doi: 10.1107/S2053229614024218.

Sheldrick, G.M. (2015b). SHELXT-Integrated space-group and crystal-structure determination. Acta Cryst. A71 71(3).

**
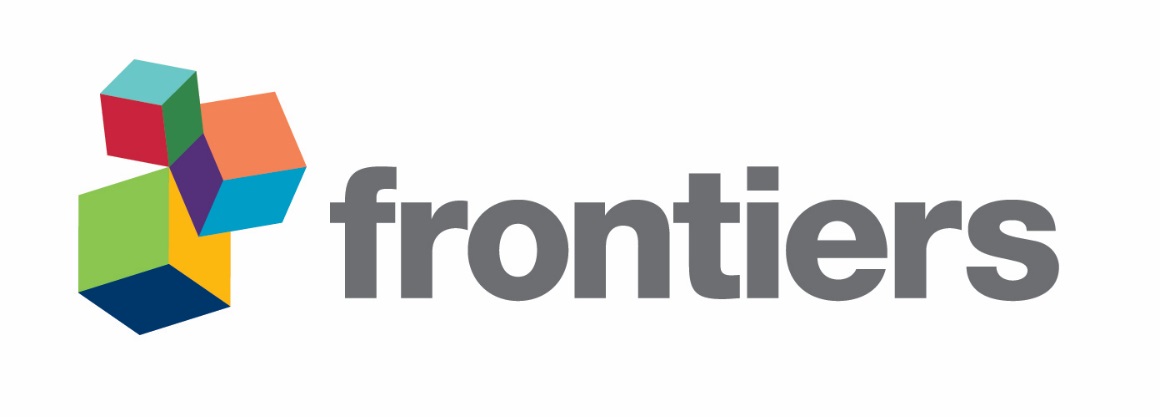
**
